# A Pyridine Dearomatization Approach for the Gram Scale Synthesis of (±)-Sparteine

**DOI:** 10.1021/acs.orglett.3c03242

**Published:** 2023-11-10

**Authors:** Pik Hoi Lam, Jeff K. Kerkovius, Sarah E. Reisman

**Affiliations:** † Division of Chemistry and Chemical Engineering, California Institute of Technology, Pasadena, California 91125, United States

## Abstract

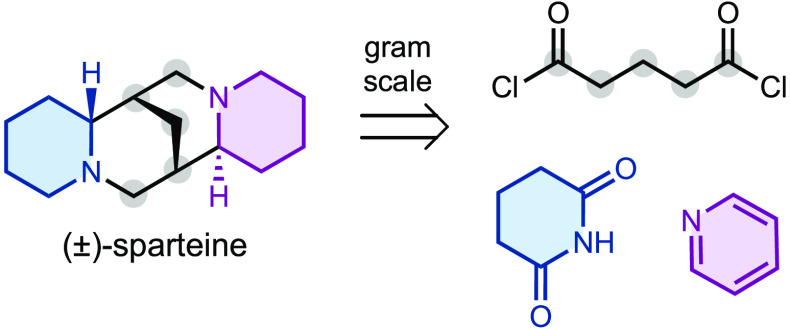

Both enantiomers of sparteine have suffered from pricing and supply chain variability, which has inspired efforts toward efficient chemical synthesis. Here, we build upon our reported synthesis of the matrine-type lupin alkaloids in order to synthesize (±)-sparteine. Specifically, selective quenching of the cyclization between glutaryl chloride and pyridine with methanol provides a functionalized quinolizidine core that was elaborated to (±)-sparteine in six additional steps on gram scale. This synthesis provides a scalable route to sparteine from inexpensive commodity chemicals utilizing a dearomative cyclization. In addition, this route provides concise access to (±)-lupinine.

Sparteine (**1**, Scheme 1) is a unique quinolizidine alkaloid first isolated from *Lupinus Barbiger* S. (Watson) in 1932.^1^ Biological studies have determined that **1** exhibits antiarrhythmic activities;^2^ however, it is better known as a chiral ligand for organic synthesis, especially for enantioselective lithiation.^3^ The cage-like conformation of **1** spatially positions both nitrogen lone pairs inward allowing η^2^-coordination to a variety of metals.^4^ The ability of sparteine to coordinate alkyl lithium reagents and generate configurationally stable anions was first developed in the 1990s and is still commonly leveraged in modern total syntheses.^5,6^ Despite its broad use and ready extraction from *Lupinus* plants, periodic supply chain issues have motivated the development of synthetic approaches to access sparteine or sparteine surrogates.^7^ Here we report a concise gram-scale synthesis of (±)-sparteine from the commodity chemicals pyridine and glutaryl chloride.

**Scheme 1:**
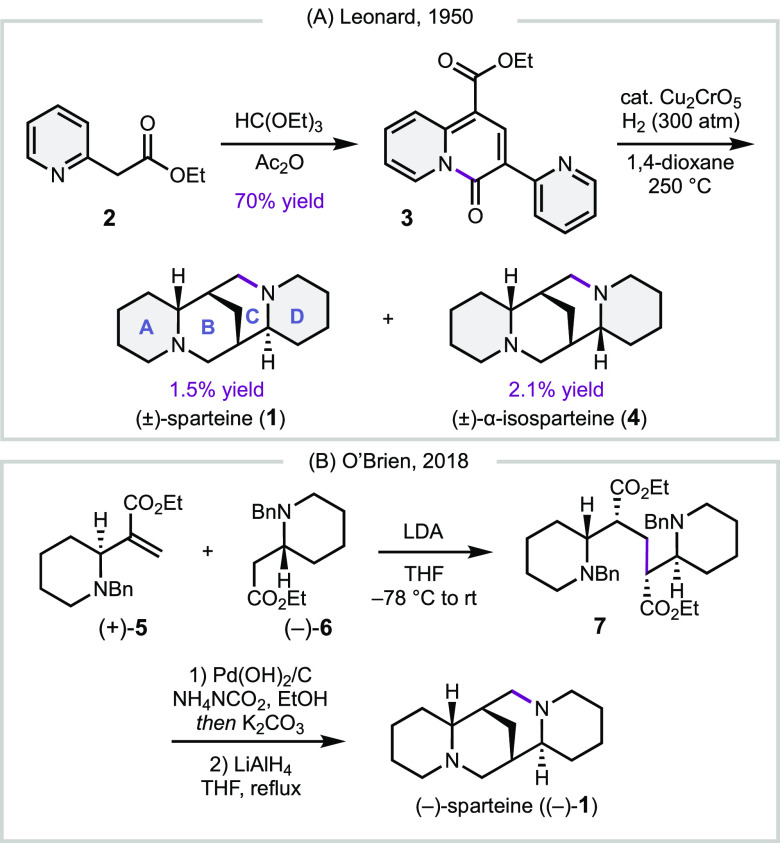
(a) First Synthesis of (±)-Sparteine; (b) First Enantioselective Synthesis of (−)-Sparteine

To date, over 20 total syntheses of sparteine have been reported.^8^ More than half of these strategies involve forming the B and C rings through an *N*-alkylation via lactamization, Mannich, or Appel reaction. The shortest synthesis was accomplished in 1950 by Leonard and co-workers (Scheme 1a),^9^ and featured an innovative exhaustive hydrogenation of pyridine-quinolizone **3**. Although the hydrogenation proceeded in a low yield, this approach provided access to (±)-**1** in only two steps from ethyl 2-(pyridin-2-yl)acetate (**2**). The first asymmetric gram-scale synthesis of sparteine was accomplished in 2018 by O’Brien (Scheme 1b).^7a^ Enzymatic resolution of the racemic ester (±)-**6** provided, after re-esterification, (+)-**6** in >99% ee. Condensation of ester (+)-**6** with 1 equiv of formaldehyde gave unsaturated ester (+)-**5**, which underwent 1,4-addition with the enolate of ester (−)-**6** to give diester **7**. Benzyl deprotection and one-pot lactamization, followed by global reduction, gave access to (−)-**1** in a total of ten steps.

We recently reported the synthesis of isomatrine, matrine, and additional matrine alkaloids using a dearomative annulation reaction of pyridine (**10**). Mechanistic studies revealed that the cascade cyclization between **10** and glutaryl chloride (**8**) occurs via a stepwise pathway that could be interrupted by addition of methanol (Scheme 2a).^10,11^ We hypothesized that if instead of methanol, intermediate **11** could be trapped with 4-pyridone (**12**), subsequent enolization and conjugate addition could form the sparteine tetracycle (Scheme 2b). Unfortunately, initial attempts to trap acid chloride **11** (formed *in situ* by the reaction of **8** with **10**) with 2- or 4-pyridone led predominately to *O*-acylation. When 4-trimethylsiloxy pyridine (**14**) was used as a protected pyridone equivalent, a product assigned by ^1^H NMR as *N*-acylated pyridone **15** could be formed in 40% yield (Scheme 2c); however, attempts to purify **15** resulted in significant hydrolysis to the corresponding carboxylic acid. The reaction of acyl chloride **11** with piperidine was also investigated, anticipating that a late-stage oxidation/enolate addition could allow elaboration to **1**. However, the acylation of piperidine with acid chloride **11** proceeded in low yields with challenging purification. Due to the challenges encountered directly leveraging acid chloride **11**, we decided to capitalize on the high yielding methanol quench/partial reduction to form **9** (Scheme 3), which we envisioned could be engaged with an appropriate pyridine surrogate at a later stage.

**Scheme 2:**
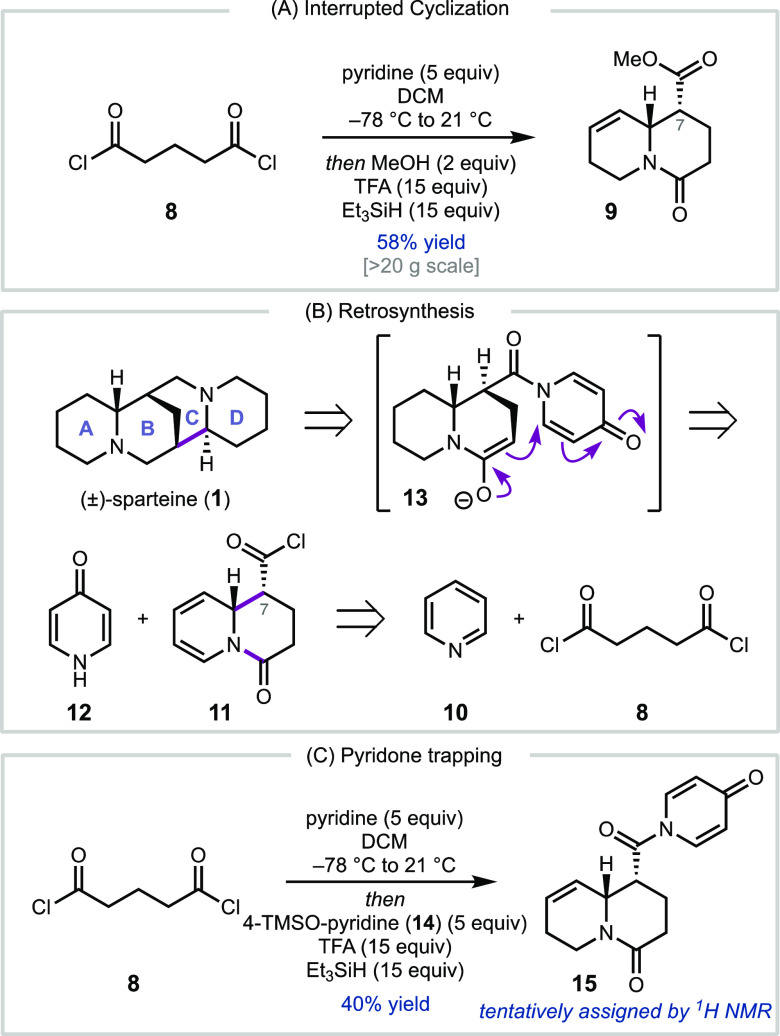
(a) Key Precedent for the Interrupted Cascade; (b) Initial Retrosynthetic Analysis; (c) Investigation of Pyridine Trapping

**Scheme 3:**
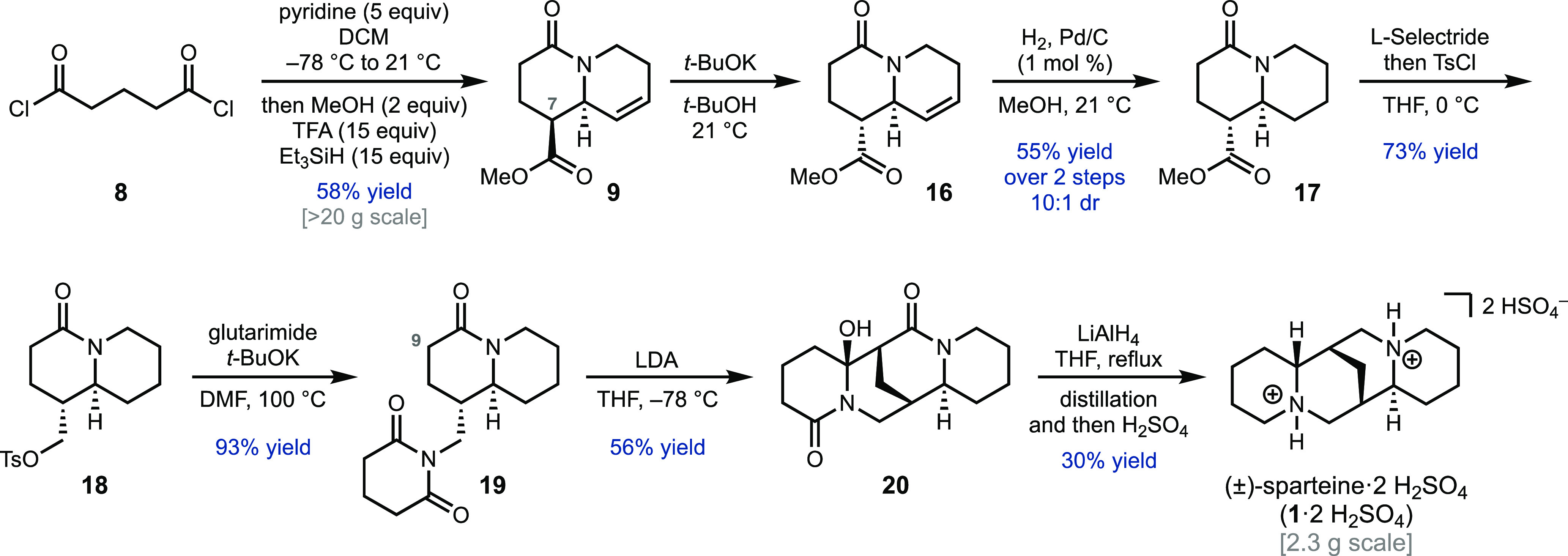
Gram Scale Total Synthesis of (±)-Sparteine

Although our initial plan called for forming the sparteine C ring through the cyclization of **13**, based on related synthetic work from Gray and Gallagher,^11^ our revised approach pursued a route involving epimerization at C7 and cyclization to form the B ring (Scheme 3). The use of *t*-BuOK in *t-*BuOH was found to be optimal for this task, giving the more thermodynamically stable *trans* isomer **16** with a 10:1 dr. The crude material was hydrogenated using catalytic Pd/C (1 mol %) to yield **17** in 55% yield over two steps. Removal of the minor epimer was difficult at this stage, so the material was advanced as a mixture of diastereomers. Chemoselective reduction of the ester of **17** with l-selectride and direct quenching of the reaction mixture with tosyl chloride provided primary tosylate **18** in 73% yield. S_N_2 reaction between tosylate **18** and glutarimide provided the *N*-alkylation product **19** in 93% yield.

Initial attempts to convert **19** to **20** by formation of the C9 enolate and intramolecular addition to glutarimide were unsuccessful. For example, treatment with LDA followed by warming to room temperature and stirring overnight resulted in complex reaction mixtures with only trace product formation. It was discovered that by decreasing the reaction temperature to −78 °C side product formation was suppressed; however, low yields of the desired product were still obtained when standard aqueous workup conditions were used. Upon further investigation it was discovered that the cyclization was very fast and decreasing the reaction time to 2 min provided significantly enhanced yields with cleaner reaction profiles. Allowing the reaction to warm up at all, even during the quenching process, led again to significant byproduct formation. Ultimately, it was determined that quenching at −78 °C with a solution of acetic acid in THF was crucial to minimize byproduct formation and product degradation. The optimized procedure was found to be scalable and could deliver gram quantities of **20** in 56% yield. Reduction of **20** with LiAlH_4_ provided (±)-**1** on a gram scale, which could be purified by distillation (56% yield (±)-**1** by qNMR) and then recrystallized as the bis-hydrogen sulfate salt from ethanol (30% yield of (±)-**1**·2H_2_SO_4_).

In addition to sparteine, ester **9** also provides access to the lupin alkaloid lupinine (**21**) (Scheme 4). In this case, direct hydrogenation of **9** provided the diastereomeric ester of **17**. Further reduction with LiAlH_4_ then provided (±)-**21** in three steps from pyridine.

**Scheme 4:**
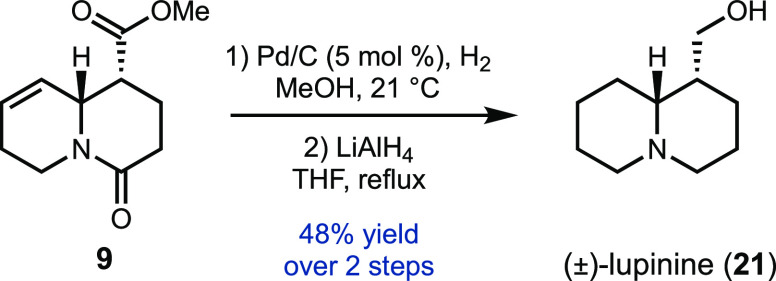
Total Synthesis of (±)-Lupinine (**21**)

In summary, the total syntheses of sparteine and lupinine have been completed in seven and three steps, respectively, from pyridine. Key to both syntheses is the interrupted dearomative cascade cyclization between pyridine and glutaryl chloride to yield quinolizidine **9**. A two-step reduction sequence provides a concise route to lupinine, while a key intramolecular enolate addition provides the carbocyclic scaffold of sparteine. This synthesis highlights the power of a dearomative approach toward complex alkaloids and can provide (±)-**1** on gram scale.

## Data Availability

The data underlying this study are available in the published article and its Supporting Information.

## Supporting Information Available

Experimental procedures, characterization data (^1^H and ^13^C NMR) for all new compounds (PDF)
