# Supplementary material for: A Pyridine Dearomatization Approach for the Gram Scale Synthesis of (±)-Sparteine
Source: Org Lett. 2023 Nov 10;25(46):8230–3. doi: 10.1021/acs.orglett.3c03242 (PMC10683365; doi:10.1021/acs.orglett.3c03242)

Supplementary Materials for

A Pyridine Dearomatization Approach for the Gram Scale Synthesis of (±)-Sparteine

Pik Hoi Lam,[1] Jeff K. Kerkovius,[1] Sarah E. Reisman[1*]

[1]Division of Chemistry and Chemical Engineering, California Institute of Technology, Pasadena, CA 91125, USA.

*Correspondence to: reisman@caltech.edu

Table of Contents

1. General Procedures……………………………………………………………………S2

2. Synthetic Procedures…………………………………………………………..…………S4

3. Notes and References……………………………………………………………….…….S18

4. ^{1}H and ^{13}C Spectral Data …...……………………………………………………...…S19

General Procedures

Unless otherwise stated, reactions were performed under an inert atmosphere (dry N_2) using freshly dried solvents and standard Schlenk techniques. Glassware was oven-dried at 120 °C for a minimum of four hours. Tetrahydrofuran (THF), methylene chloride (DCM), acetonitrile (ACN), methanol (MeOH), benzene (PhH), and toluene (PhMe) were dried by passing through activated alumina columns. CH_2Cl_2 (D150-4), benzene (PhH, OmniSolv, BX0212-1), acetonitrile (A998-4), pentane (P399-4), acetone (A18-20), hexanes (H292-20), and *n*-butanol (A399-4) were purchased from Fisher and used as received. Anhydrous *N*,*N*-dimethylformamide (DMF) was purchased from VWR (EM-DX1727-6) and used as received. All reactions were monitored by thin-layer chromatography using EMD/Merck silica gel 60 F254 pre-coated plates (0.25 mm) and were visualized by UV or by staining with *p*-anisaldehyde or potassium permanganate ($KMnO_4$). Flash column chromatography was performed as described by Still et al.[1] using silica gel (particle size 0.032–0.063) purchased from MilliporeSigma. ^{1}H and ^{13}C NMR spectra were recorded on a Bruker Avance III HD with Prodigy cryoprobe (at 400 MHz and 101 MHz, respectively), a Varian Inova 500 (at 500 MHz and 126 MHz, respectively), a Bruker 400 MHz Spectrometer with broadband iProbe, or a Varian Inova 600 (at 600 MHz and 150 MHz, respectively), and are reported relative to internal $CDCl_3$ (^{1}H, δ = 7.26; ^{13}C, δ = 77.16) or CD_2Cl_2 ^{1}H, δ = 5.32; ^{13}C, δ = 53.84). $CDCl_3$ was stored over anhydrous potassium carbonate (K_2CO_3). Data for ^{1}H NMR spectra are reported as follows: chemical shift (δ ppm) (multiplicity, coupling constant (Hz), integration). Multiplicity and qualifier abbreviations are as follows: s = singlet, d = doublet, t = triplet, q = quartet, m = multiplet, br = broad. IR spectra were recorded on a Perkin Elmer Paragon 1000 spectrometer and are reported in frequency of absorption (cm^{-1}). HRMS data were acquired using an Agilent 6230 Series time-of-flight (TOF) mass spectrometer with an Agilent G1978A ion trap or by LC-MS using a Waters LCT Premier XE Electrospray TOF mass spectrometer interfaced with Waters UPLC chromatography, or by GC-MS interfaced with a JEOL JMS-T2000 GC AccuTOF GC-Alpha with Field Ionization. Molecular formulas of the compounds [M] are given, with the observed ion fragment in brackets, e.g. $[M+H]^+$. Melting points were determined using a Büchi B-545 capillary melting point apparatus, and the values reported are uncorrected. Unless otherwise stated, chemicals and reagents were used as received. Reagents were purchased from commercial vendors as follows: Solid potassium *tert*-butoxide was purchased from STREM

Chemicals Inc., stored in a glovebox, and used as received. Glutaryl chloride was purchased from Oakwood Chemicals Inc. and was used as received. Anhydrous pyridine, and palladium on carbon (10%) were purchased from MilliporeSigma and were used as received. ^{1}H qNMR standards trimethylphenyl silane (99% purity) and pyrazine (≥99% purity) were purchased from MilliporeSigma and were used as received.

Preparation of methyl ester 9:

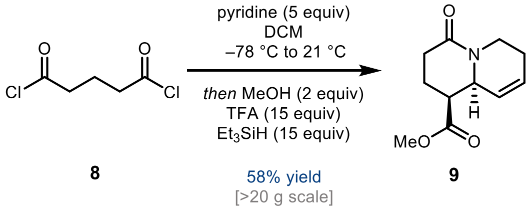

A 3-neck 3 L oven dried N_2 flushed round bottom flask was equipped with an overhead stirrer, a thermocouple, and a rubber septum. The flask was charged with glutaryl chloride **8** (22.8 mL, 178 mmol, 1.0 equiv) and DCM (1.8 L, 0.1 M). The solution was cooled to –50 °C and then pyridine (71.8 mL, 888 mmol, 5.0 equiv) was added dropwise at such a rate as to prevent the temperature from increasing above –40 °C. The thick slurry was stirred at –50 °C for 15 minutes and then allowed to warm to ambient temperature. As soon as the reaction became homogenous (*ca.* 30-60 minutes) methanol (36.0 mL, 888 mmol, 5.0 equiv) was added. The solution was cooled to 0 °C and then triethylsilane (425 mL, 2.66 mol, 15 equiv) added, followed by a dropwise addition of trifluoroacetic acid (204 mL, 2.66 mmol, 15 equiv) at such a rate as to prevent the temperature from increasing above 20 °C (*ca.* 15 minutes). Upon completion of the addition, the reaction was allowed to warm to 21 °C and stir for 18 hours. Once complete, the reaction was quenched with sat. Na_2CO_3, and the mixture was extracted with DCM (3 x 250 mL). The combined organic layers were dried over anhydrous Na_2SO_4, filtered, and concentrated under reduced pressure. The crude product was purified via SiO_2 column chromatography [1900 g SiO_2, 120 mm diameter column, eluted with 50% Acetone/50% Hexanes] to yield the alkenyl methyl ester **9** as a pale yellow crystalline solid (21.6 g, 58% yield).

(±)-Methyl Ester 9:

^{1}H NMR (500 MHz, $CDCl_3$): δ 6.04 – 5.89 (m, 1H), 5.50 (ddt, *J* = 10.1, 2.9, 1.4 Hz, 1H), 4.76 (ddt, *J* = 12.8, 5.8, 1.3 Hz, 1H), 4.37 (ddt, *J* = 6.9, 4.9, 2.4 Hz, 1H), 3.69 (s, 3H), 3.01 (dt, *J* = 6.6, 4.9 Hz, 1H), 2.73 (td, *J* = 12.2, 4.2 Hz, 1H), 2.61 (ddd, *J* = 17.7, 7.8, 6.7 Hz, 1H), 2.43 (dt, *J* = 17.6, 6.5 Hz, 1H), 2.33 (ddtd, *J* = 20.3, 11.9, 6.0, 2.5 Hz, 1H), 2.10 – 1.96 (m, 3H).

^{13}C NMR (101 MHz, $CDCl_3$): δ 172.0, 169.1, 129.1, 125.9, 55.8, 52.0, 43.1, 39.8, 30.4, 24.7, 21.6.

FTIR (NaCl, thin film): 3031, 2951, 2841, 1736, 1642, 1459, 1436, 1417, 1280, 1263, 1233, 1193, 1163, 1014, 988, 917 cm^{-1}.

HRMS: (FI-TOF) m/z: [$M+H^+$] calc'd for $C_{11}H_{15}NO_3H^+$ 209.1046, found 209.1048.

TLC (50% acetone/50% hexanes), **R_f:** 0.23 ($KMnO_4$).

M.P. 38.4 – 40.6 °C.

Preparation of methyl ester S1:

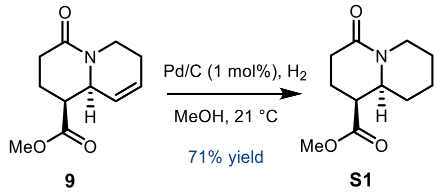

A 25 mL flask was charged with alkenyl methyl ester **9** (100 mg, 0.478 mmol, 1.0 equiv), 10% palladium on carbon (25.4 mg, 23.9 μmol, 5 mol %) and methanol (4.78 mL, 0.1 M). The flask was purged with N_2 (balloon) then with H_2 (balloon). The reaction was stirred vigorously (1500 rpm) until complete consumption of the starting material was observed by TLC (*ca.* 3 hours). Upon completion, the reaction was filtered over celite, concentrated under reduced pressure, and purified via SiO_2 column chromatography [20 g SiO_2, 20 mm column diameter, eluted with 25% acetone/75% hexanes] to yield the ester **S1** as a white crystalline solid (72.2 mg, 71% yield).

(±)-Methyl Ester 16:

^{1}H NMR (500 MHz, $CDCl_3$): δ 4.76 (ddt, *J* = 12.8, 4.3, 2.0 Hz, 1H), 3.73 (s, 3H), 3.74 – 3.67 (m, 1H), 2.98 (dt, *J* = 11.0, 5.8 Hz, 1H), 2.60 – 2.44 (m, 2H), 2.40 – 2.28 (m, 1H), 2.09 – 1.98 (m, 2H), 1.95 (d, *J* = 13.6 Hz, 1H), 1.64 (ddd, *J* = 10.7, 8.4, 5.5 Hz, 1H), 1.60 – 1.52 (m, 1H), 1.51 – 1.36 (m, 3H).

^{13}C NMR (101 MHz, $CDCl_3$): δ 172.2, 167.9, 58.1, 52.2, 44.7, 43.5, 31.5, 28.4, 25.5, 25.0, 19.6.

FTIR (NaCl, thin film): 2985, 2952, 1738, 1635, 1439, 1420, 1275, 1262, 1168 cm^{-1}.

HRMS: (FI-TOF) m/z: [$M+H^+$] calc'd for $C_{11}H_{17}NO_3H^+$ 211.1203, found 211.1207.

TLC (25% acetone/75% hexanes), **R_f:** 0.22 ($KMnO_4$).

M.P. 74.6 – 75.9 °C.

Preparation of methyl ester 16:

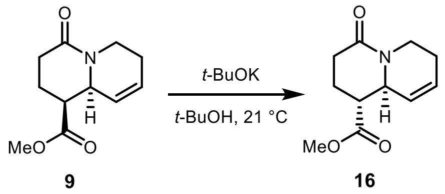

A 1 L N_2 -flushed flask was charged with **9** (21.6 g, 103 mmol, 1.0 equiv) and *t*-BuOH (413 mL, 0.25 M). Next, potassium *t*-BuOK (4.63 g, 41.3 mmol, 0.4 equiv) was added in a single portion. The solution was stirred at 21 °C until a 10:1 ratio of **16**:**9** was reached as judged by ^{1}H NMR aliquots (*ca.* 4 hours, $CDCl_3$). Upon completion, the reaction was quenched with acetic acid (2.36 mL, 41.3 mmol, 0.4 equiv). The reaction was concentrated under reduced pressure. The residue was diluted with sat. aq. $NaHCO_3$ and was extracted with DCM (3 x 100 mL). The combined organic layers were dried over anhydrous Na_2SO_4, filtered, and concentrated under reduced pressure. The crude product was used directly in the next step without additional purification. An analytically pure sample was obtained by SiO_2 column chromatography (20 g SiO_2, 20 mm column, 40% Acetone/60% Hexanes) to provide **16** as a white crystalline solid (19.1 g, 88% yield).

(±)-Methyl Ester 16:

^{1}H NMR (400 MHz, $CDCl_3$): δ 5.94-5.90 (m, 1H), δ5.56-5.50 (m, 1H), 4.83 (ddt, *J* = 12.9, 5.8, 1.4 Hz, 1H), 4.31 (dq, *J* = 10.6, 2.1 Hz, 1H), 3.76 (s, 3H), 2.64 (td, *J* = 12.4, 4.1 Hz, 1H), 2.56 (ddd, *J* = 17.7, 5.5, 2.5 Hz, 1H), 2.47 (ddd, *J* = 12.3, 10.6, 3.2 Hz, 1H), 2.40 (ddd, *J* = 18.1, 12.6, 6.3 Hz, 1H), 2.25 (dddq, *J* = 17.7, 11.9, 5.9, 2.6 Hz, 1H), 2.11 – 2.00 (m, 2H), 1.94 (qd, *J* = 12.6, 5.4 Hz, 1H).

^{13}C NMR (101 MHz, $CDCl_3$): δ 173.6, 168.1, 127.9, 127.1, 56.6, 52.7, 46.4, 39.0, 31.8, 25.3, 24.2

FTIR (NaCl, thin film): 3052, 2953, 1728, 1639, 1434 cm^{-1}.

HRMS: (ESI-TOF) m/z: [M+H$^+$] calc'd for $C_{11}H_{15}O_3NH^+$ 210.1124, found 210.1124.

TLC: 50% acetone in 50% hexane, **R_f** = 0.44 ($KMnO_4$).

Preparation of methyl ester 17:

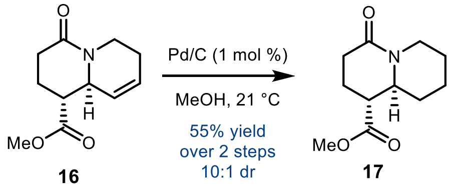

A 1 L flask was charged with all the trans methyl ester **16** from the previous step (*ca.* 21.6 g, 103 mmol, 1.0 equiv), 10% palladium on carbon (971 mg, 0.91 mmol, 1 mol %), and MeOH (456 mL, 0.2 M). The flask was purged with N_2 followed by H_2 then the reaction was stirred at 1500 RPM at 21 °C until full consumption of the starting material was observed by TLC (*ca.* 2 hours). Upon completion, the solution was then filtered through celite with DCM, concentrated under reduced pressure, and purified via SiO_2 column chromatography (1900 g SiO_2, 120 mm column, 40:60 acetone/hexane) to provide **17** as a white crystalline solid (10.6 g, 55% yield).

(±)-Methyl Ester 17:

^{1}H NMR (400 MHz, $CDCl_3$): δ 4.80 (ddt, *J* = 13.2, 4.2, 2.1 Hz, 1H), 3.73 (s, 3H), 3.58 (ddd, *J* = 11.0, 8.2, 2.5 Hz, 1H), 2.59 – 2.40 (m, 3H), 2.34 (ddd, *J* = 17.2, 11.1, 5.5 Hz, 1H), 2.05 – 1.98 (m, 1H), 1.92 (dtd, *J* = 13.2, 11.0, 4.8 Hz, 1H), 1.87 – 1.77 (m, 2H), 1.69 (ddq, *J* = 13.4, 4.0, 2.1 Hz, 1H), 1.48 (qt, *J* = 12.2, 3.4 Hz, 1H), 1.43 – 1.32 (m, 1H), 1.25 (tdd, *J* = 13.1, 11.3, 3.5 Hz, 1H).

^{13}C NMR (101 MHz, $CDCl_3$): δ 174.0, 168.4, 58.4, 52.7, 47.1, 43.1, 33.8, 31.8, 25.5, 24.7, 23.5

FTIR (NaCl, thin film): 3050, 2856, 1732, 1645, 1454 cm^{-1}.

HRMS: (ESI-TOF) m/z: [M+H^+] calc'd for $C_{11}H_{17}O_3NH^+$ 212.1281, found 212.1281.

TLC: 50% acetone in 50% hexane, $\mathbf{R_f}$ = 0.48 (Seebach's "Magic" Stain)

Preparation of (±)-tosylate 18:

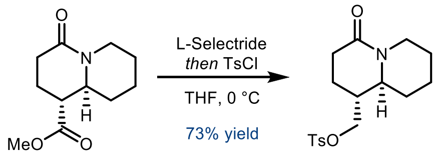

A 1 L N_2 flushed flask was charged with **17** (10.6 g, 50.2 mmol, 1.0 equiv) and THF (201 mL, 0.25 M). The solution was cooled to 0 °C and then L-Selectride (1 M in THF, 105 mL, 105 mmol, 2.1 equiv) was added over the course of 5 minutes. The reaction was allowed to stir for 10 mins at 0 °C after which *p*-toluenesulfonyl chloride (16.3 g, 85.3 mmol, 1.7 equiv) was added as a solution in THF (25 mL, 1 M) at such a rate as to prevent the temperature from increasing above 10 °C (*ca.* 10 minutes). The clear solution was stirred for 15 minutes, after which it was quenched by the dropwise addition of a mixture of hydrogen peroxide (30% in water, 9.2 mL, 90.1 mmol, 1.8 equiv) and sodium hydroxide (4.0 g, 100 mmol, 2.0 equiv). The reaction mixture was stirred at 0 °C for 1 hour. Upon the completion, the reaction mixture was concentrated under reduced pressure, diluted with sat. NH_4Cl (50 mL) and extracted with DCM (3 x 75 mL). The combined organic layers were dried over anhydrous Na_2SO_4, filtered, and concentrated under reduced pressure. The crude product was purified via column chromatography (1500 g SiO_2, 120 mm column, 40:60 Acetone/Hexane) to yield **18** in a white crystalline solid (12.4 g, 73% yield).

(±)-Tosylate 18:

^{1}H NMR (400 MHz, $CDCl_3$): δ 7.78 (d, *J* = 8.2 Hz, 2H), 7.37 (d, *J* = 8.0 Hz, 2H), 4.75 (ddt, *J* = 13.1, 4.2, 2.2 Hz, 1H), 4.30 – 3.74 (m, 2H), 3.04 (ddd, *J* = 11.5, 7.3, 2.5 Hz, 1H), 2.46 (s, 3H) 2.24 (ddd, *J* = 17.4, 10.3, 5.4 Hz, 2H), 2.24 (ddd, *J* = 17.4, 10.3, 5.4 Hz, 1H), 1.88 – 1.74 (m, 4H), 1.61 (dddd, *J* = 20.4, 15.2, 10.2, 3.7 Hz, 2H), 1.46 – 1.30 (m, 2H), 1.27 – 1.16 (m, 1H).

^{13}C NMR (101 MHz, $CDCl_3$): δ 168.6, 145.7, 133.0, 130.5, 128.4, 71.1, 58.3, 43.5, 40.0, 33.5, 31.1, 25.5, 24.9, 22.1.

FTIR (NaCl, thin film): 3053, 2942, 1633, 1362, 1265 cm^{-1}.

HRMS: (ESI-TOF) m/z: [M+H$^+$] calc'd for $C_{17}H_{23}SO_4NH^+$ 338.1424, found 338.1421.

TLC: 50% acetone in 50% hexane, $\mathbf{R_f}$ = 0.36 ($KMnO_4$)

Preparation of (±)-glutarimide 19:

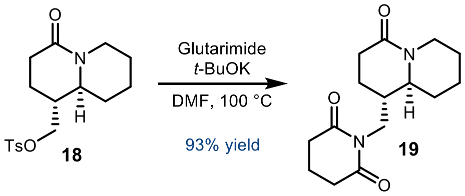

An oven dried N_2 flushed 250 mL flask was charged with glutarimide (9.56 g, 84.5 mmol, 2.3 equiv) and DMF (184 mL, 0.2 M). To the solution was added *t*-BuOK (5.77 g, 51.4 mmol, 1.4 equiv) after which the mixture was allowed to stir for 15 minutes. A separate oven dried N_2 flushed 500 mL flask was charged with tosylate **18** (12.4 g, 36.7 mmol, 1.0 equiv) and DMF (92 mL, 0.4 M). The glutarimide solution was cannulated into the tosylate solution over the course of 5 minutes. The reaction mixture was then heated at 100 °C in an oil bath until complete consumption of the starting material was observed by TLC (*ca.* 3 hours). Upon completion, the reaction mixture was cooled to 21 °C. The flask was equipped with a short-path distillation head, and the majority of the DMF was removed by distillation under reduced pressure (35 °C, 0.3 torr). The residue was diluted in sat. $NaHCO_3$ (100 mL) and was extracted with DCM (3 x 75 mL). The combined organic layers were dried over anhydrous Na_2SO_4, filtered, and concentrated under reduced pressure. The crude product was purified by SiO_2 column chromatography (500 g SiO_2, 80 mm column, 50% Acetone/50% Hexanes) to yield the product **19** as a white crystalline solid (9.50 g, 93% yield).

(±)-Glutarimide 19:

^{1}H NMR (400 MHz, $CDCl_3$): δ 4.81 (ddt, *J* = 13.2, 4.3, 2.2 Hz, 1H), 4.15 – 3.55 (m, 2H), 3.00 (ddd, *J* = 10.5, 7.5, 2.4 Hz, 1H), 2.69 (t, *J* = 6.5 Hz, 4H), 2.48 (dt, *J* = 17.3, 5.0 Hz, 1H), 2.38 (td, *J* = 12.9, 2.8 Hz, 1H), 2.18 (ddd, *J* = 16.8, 10.7, 5.3 Hz, 1H), 2.03 – 1.92 (m, 3H), 1.90 – 1.80 (m, 2H), 1.65 (ddt, *J* = 13.7, 9.3, 4.6 Hz, 2H), 1.55 – 1.34 (m, 3H), 1.29 (qd, *J* = 12.7, 3.6 Hz, 1H).

^{13}C NMR (101 MHz, $CDCl_3$): δ 173.2, 169.1, 60.9, 43.5, 42.3, 39.4, 33.8, 33.6, 31.5, 25.7, 25.2, 23.2, 17.6.

FTIR (NaCl, thin film): 3053, 2943, 1679, 1631, 1264 cm^{-1}.

HRMS: (ESI-TOF) m/z: [M+H$^+$] calc'd for $C_{15}H_{22}O_3N_2H^+$ 279.1705, found 279.1703.

TLC: 50% acetone in 50% hexane, **R_f** = 0.48 (Seebach's "Magic" Stain)

Preparation of (±)-bis-amide 20:

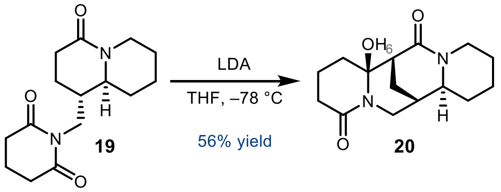

A 250 mL oven dried N_2 flushed flask was charged with diisopropylamine (8.61 mL, 61.4 mmol, 1.8 equiv) and THF (68 mL, 1.0 M). The solution was cooled to 0 °C then *n*-buthyllithium (2.5 M in hexanes, 24.6 mL, 61.4 mmol, 1.8 equiv) was added dropwise after which the reaction was allowed to stir for 15 minutes at 0 °C. A separate 500 mL oven dried N_2 flushed flask was charged with glutarimide **19** (9.50 g, 34.1 mmol, 1.0 equiv) and THF (341 mL, 0.1 M) and then cooled to –78 °C. The LDA solution was cannulated into the glutarimide solution at –78 °C rapidly over the course of 2 minutes. The reaction was stirred for an additional 2 minutes at –78 °C and then quenched with acetic acid (9.8 mL, 171 mmol, 5.0 equiv) at –78 °C. The reaction mixture was removed from the cooling bath and was concentrated under reduced pressure. The crude reaction mixture was diluted in sat. NH_4Cl (100 mL) and was extracted with DCM (3 x 75 mL). The combined organic layers were dried over anhydrous Na_2SO_4, filtered, and concentrated under reduced pressure. The crude product was purified via SiO_2 column chromatography (500 g SiO_2, 80 mm column, 15% MeOH/85% EtOAc) to yield the product **20** as a white crystalline solid (5.30 g, 56% yield).

(±)-Bis-amide 20:

^{1}H NMR (400 MHz, $CDCl_3$): δ 4.93 (dd, *J* = 14.2, 10.7 Hz, 1H), 4.74 (ddt, *J* = 13.1, 4.2, 1.9 Hz, 1H), 4.07 (s, 1H), 3.02 – 2.97 (m, 1H), 2.72 (dt, *J* = 4.1, 2.0 Hz, 1H), 2.57 – 2.46 (m, 3H), 2.33 (ddd, *J* = 17.6, 12.6, 6.7 Hz, 1H), 2.23 (pq, *J* = 9.2, 3.2 Hz, 2H), 1.99 – 1.90 (m, 2H), 1.88 – 1.79 (m, 2H), 1.76 – 1.66 (m, 2H), 1.66 – 1.51 (m, 3H), 1.48 – 1.36 (m, 2H).

^{13}C NMR (101 MHz, $CDCl_3$): δ 170.8, 168.9, 84.6, 62.9, 49.1, 44.4, 38.7, 38.5, 32.3, 31.8, 31.2, 26.1, 25.2, 20.4, 16.4.

FTIR (NaCl, thin film): 3053, 1641, 1615, 1269, 1407 cm^{-1}.

HRMS: (ESI-TOF) m/z: [$M+H^+$] calc'd for $C_{15}H_{22}O_3N_2H^+$ 279.1705, found 279.1703.

TLC: 15% methanol in 85% ethyl acetate, $\mathbf{R_f}$ = 0.26 (Seebach's "Magic" Stain)

Preparation of sparteine ((±)-1) and recrystallization as bis-hydrogen sulfate salt:

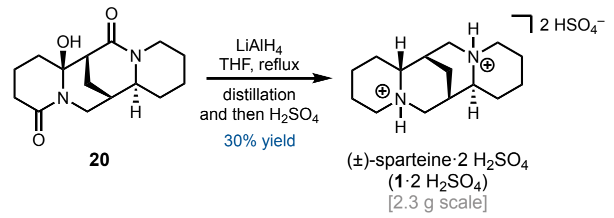

An N_2 flushed oven dried 1L round bottom flask was charged with bis-amide **20** (5.00 g, 18.0 mmol, 1.0 equiv) and THF (180 mL, 0.1 M). To the solution at 21 °C was added $LiAlH_4$ (1.0 M solution in THF, 341 mL, 341 mmol, 19 equiv). The solution was heated to reflux in an oil bath for 16 hours. Upon completion, the solution was cooled to 21 °C and then poured into a solution of sat. aq. Rochelles salt (500 mL) and ice (500 g). After quenching, 3 M NaOH (100 mL) was added, and the mixture was stirred for 15 minutes to break apart the aluminum solids into a white slurry. The mixture was concentrated under reduced pressure to remove the THF. Subsequently, the mixture was extracted with Et_2O (3 x 100 mL). The combined organic layers were washed with brine (100 mL), dried over anhydrous Na_2SO_4, filtered, and concentrated under reduced pressure. The crude residue was purified via distillation under reduced pressure (300 mTorr, 80.0 °C – 82.1 °C, 120 °C oil bath temperature) to yield 2.30 g of sparteine (**1**) (80% purity by qNMR in $CDCl_3$, pyrazine standard, 56% corrected yield). The obtained pale-yellow oil was treated with 2 M H_2SO_4 (8.0 mL, 16 mmol, 2.1 equiv) followed by freezing and water removal through lyophilization. The obtained solids were suspended in boiling EtOH (15 mL), cooled to 0 °C, and the crystals were isolated by vacuum filtration to provide sparteine bis-hydrogen sulfate as a pentahydrate, a white crystalline solid (2.50 g, 30% yield from **20**). Characterization data match previously reported data.[3]

(±)-Sparteine (1):

^{1}H NMR (600 MHz, $CDCl_3$): δ 4.93 (dd, *J* = 14.2, 10.7 Hz, 1H), 4.74 (ddt, *J* = 13.1, 4.2, 1.9 Hz, 1H), 4.07 (s, 1H), 3.02 – 2.97 (m, 1H), 2.72 (dt, *J* = 4.1, 2.0 Hz, 1H), 2.57 – 2.46 (m, 3H), 2.33 (ddd, *J* = 17.6, 12.6, 6.7 Hz, 1H), 2.23 (pq, *J* = 9.2, 3.2 Hz, 2H), 1.99 – 1.90 (m, 2H), 1.88 – 1.79 (m, 2H), 1.76 – 1.66 (m, 2H), 1.66 – 1.51 (m, 3H), 1.48 – 1.36 (m, 2H).

^{13}C NMR (101 MHz, $CDCl_3$): δ 170.8, 168.9, 84.6, 62.9, 49.1, 44.4, 38.7, 38.5, 32.3, 31.8, 31.2, 26.1, 25.2, 20.4, 16.4.

FTIR (NaCl, thin film): 2985, 2933, 1421, 1268 cm^{-1}.

FTIR (ATR, diamond): 3045, 2956, 1195, 1013, 829 cm^{-1}

HRMS: (ESI-TOF) m/z: $[M+H^+]$ calc'd for $C_{15}H_{27}N_2H^+$ 235.2169, found 235.2177.

TLC: 15% methanol in 85% ethyl acetate, $\mathbf{R_f}$ = 0.26 (Seebach's "Magic" Stain)

(±)-Sparteine·bis-hydrogen sulfate salt (1·2 H_2SO_4):

^{1}H (600 MHz, D_2O): δ 3.66 (dd, J = 14.5, 11.3 Hz, 1H), 3.52 – 3.45 (m, 3H), 3.41 (dt, J = 12.1, 3.3 Hz, 1H), 3.31 (dd, J = 11.9, 2.5 Hz, 1H), 3.20 – 3.14 (m, 2H), 3.11 (td, J = 12.7, 3.5 Hz, 2H), 2.62 (d, J = 11.3 Hz, 1H), 2.30 (s, 1H), 2.15 (dq, J = 15.3, 4.1 Hz, 1H), 2.04 (d, J = 14.7 Hz, 1H), 1.98 – 1.86 (m, 6H), 1.86 – 1.71 (m, 3H), 1.71 – 1.56 (m, 3H).

^{13}C NMR (101 MHz, D_2O): δ 66.5, 63.2, 57.1, 56.5, 55.0, 48.9, 32.0, 31.3, 29.1, 26.8, 22.7, 22.5, 22.4, 21.9, 21.5

HRMS: (ESI-TOF) m/z: $[M^{2+}]$ calc'd for $C_{15}H_{28}N_2^{2+}$ 118.1121, found 118.1123.

Table S1. *^{1}H NMR data for authentic vs synthetic (±)-sparteine bis-hydrogen sulfate salt (D_2O).*

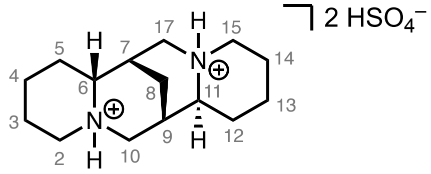

(±)-**1**·2 H_2SO_4 literature δ ppm (400 MHz, D_2O)[3]	(±)-**1**·2 H_2SO_4 recorded δ ppm (600 MHz, D_2O)
3.59 (br t, *J* = 13.0 Hz, 1H)	3.66 (dd, *J* = 14.5, 11.3 Hz, 1H)
3.48–3.31 (m, 4H)	3.52 – 3.45 (m, 3H)
–	3.41 (dt, *J* = 12.1, 3.3 Hz, 1H)
3.24 (br d, *J* = 11.0 Hz, 1H)	3.31 (dd, *J* = 11.9, 2.5 Hz, 1H)
3.17–2.96 (m, 4H)	3.20 – 3.14 (m, 2H)
–	3.11 (td, *J* = 12.7, 3.5 Hz, 2H)
2.55 (br d, *J* = 10.0 Hz, 1H)	2.62 (d, *J* = 11.3 Hz, 1H)
2.22 (br s, 1H)	2.30 (s, 1H)
–	2.15 (dq, *J* = 15.3, 4.1 Hz, 1H)
2.08 (br d, *J* = 15.0 Hz, 1H)	2.04 (d, *J* = 14.7 Hz, 1H)
2.01–1.44 (m, 13H)	1.98 – 1.86 (m, 6H)
–	1.86 – 1.71 (m, 3H)
–	1.71 – 1.56 (m, 3H)

Table S2. *^{13}C NMR data for authentic vs synthetic (±)-sparteine bis-sulfate (D_2O).*

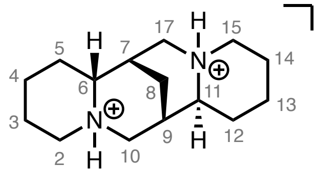

Carbon Number	(±)-**1**·2 H_2SO_4 literature δ ppm (101 MHz, D_2O)[3]	(±)-**1**·2 H_2SO_4 recorded δ ppm (101 MHz, D_2O)	Δ δ
6	66.5	66.5	0.0
11	63.2	63.2	0.0
10	57.2	57.1	–0.1
2	56.5	56.5	0.0
15	55.0	55.0	0.0
17	49.0	48.9	–0.1
9	32.0	32.0	0.0
12	31.3	31.3	0.0
7	29.1	29.1	0.0
5	26.8	26.8	0.0
3	22.7	22.7	0.0
4	22.5	22.5	0.0
14	22.4	22.4	0.0
8	21.9	21.9	0.0
13	21.5	21.5	0.0

Preparation of (±)-lupinine (21):

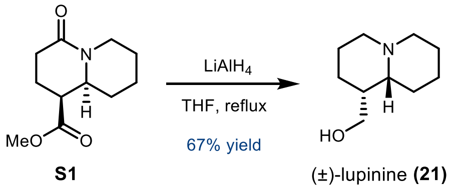

An oven dried N_2 flushed 25 mL flask was charged with methyl ester **S1** (50.0 mg, 237 μmol, 1.0 equiv) and THF (2.4 mL, 0.1 M) after which the flask was equipped with a reflux condenser that had been purged with N_2. The solution was heated to reflux in an oil bath and then lithium aluminum hydride (1.0 M in THF, 1.32 mL, 1.32 mmol, 5.6 equiv) was added dropwise. The solution was refluxed for 3 hours under N_2. Upon completion, the reaction was cooled to ambient temperature, and quenched by a dropwise addition of a saturated solution of Rochelles salt (10 mL). The reaction mixture was extracted with DCM (5 x 20 mL). The combined organic layers were dried over anhydrous Na_2SO_4, filtered, and concentrated under reduced pressure. The crude product was purified via SiO_2 column chromatography [20 g SiO_2, 20 mm column diameter, eluted with 35% 2 M NH_3 in MeOH/65% ACN] to yield (±)-lupinine (**21**) as a white crystalline solid (26.9 mg, 67% yield). Characterization data match previously reported data.[2]

(±)-Lupinine (21):

^{1}H NMR (500 MHz, CDCl$_3$): δ 5.43 (s, 1H), 4.16 (ddd, J = 10.7, 4.7, 1.7 Hz, 1H), 3.69 (d, J = 10.7 Hz, 1H), 2.86 – 2.78 (m, 2H), 2.23 – 2.08 (m, 2H), 2.01 (td, J = 12.8, 3.0 Hz, 1H), 1.90 – 1.69 (m, 4H), 1.65 – 1.49 (m, 6H), 1.26 (qt, J = 13.5, 4.5 Hz, 1H).

^{13}C NMR (101 MHz, CDCl$_3$): δ 66.2, 65.2, 57.3, 57.2, 38.2, 31.6, 29.9, 25.8, 24.8, 23.1.

FTIR (NaCl, thin film): 2985, 2941, 2859, 1466, 1445, 1421, 1268, 1262 cm^{-1}.

HRMS: (FI-TOF) m/z: [M+H$^+$] calc'd for $C_{10}H_{19}NOH^+$ 169.1461, found 169.1462.

TLC (35% 2 M NH_3 in MeOH/65% ACN), **R_f:** 0.39 ($KMnO_4$).

M.P. 47.9 – 51.1 °C.

Table S3. *^{1}H NMR data for authentic vs synthetic (±)-lupinine.*

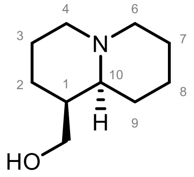

Lupinine literature δ ppm[2] (300 MHz, $CDCl_3$)	Lupinine recorded δ ppm (500 MHz, $CDCl_3$)
4.75 (br s, 1H)	5.43 (br s, 1H)
4.09 - 4.14, (m, 1H)	4.16 (ddd, *J* = 10.7, 4.7, 1.7 Hz, 1H)
3.67 (d, *J* = 10.8 Hz, 1H)	3.69 (d, *J* = 10.7, 1H)
2.81 - 2.77 (m, 2H)	2.86 - 2.78 (m, 2H)
2.14 - 1.99 (m, 3H)	2.23 - 2.08 (m, 2H)
–	2.01 (td, *J* = 12.8, 3.0 Hz, 1H)
1.74 - 1.80 (m, 4H)	1.90 - 1.69 (m, 4H)
1.58 - 1.52 (m, 6H)	1.65 - 1.49 (m, 6H)
1.30 - 1.15 (m, 1H)	1.26 (qt, *J* = 13.5, 4.5 Hz, 1H)

Table S4. *^{13}C NMR data for authentic vs synthetic (±)-lupinine.*

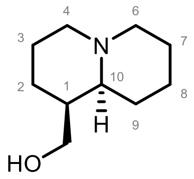

Carbon No. lupinine	Lupinine literature δ ppm (75 MHz, $CDCl_3$)	Lupinine recorded δ ppm (101 MHz, $CDCl_3$)	Δ δ
1	38.3	38.2	-0.1
2	31.3	31.6	0.3
3	23.0	23.1	0.1
4	57.0	57.2	0.2
5	57.2	57.3	0.1
6	24.5	24.8	0.3
7	25.7	25.8	0.1
8	29.8	29.9	0.1
9	65.0	65.2	0.2
10	65.9	66.2	0.3

Notes and References

(1) Still, W. C.; Kahn, M.; Mitra, A. Rapid Chromatographic Technique for Preparative Separations with Moderate Resolution. *J. Org. Chem.* **1978**, *43*, 2923–2925.

(2) Santos, L. S.; Mirabal-Gallardo, Y.; Shankaraiah, N.; Simirgiotis, M. J. Short Total Synthesis of (-)-Lupinine and (-)-Epiquinamide by Double Mitsunobu Reaction. *Synthesis* **2011**, *2011*, 51–56.

(3) Firth, J. D.; Canipa, S. J.; Ferris, L.; O'Brien, P. Gram-Scale Synthesis of the (−)-Sparteine Surrogate and (−)-Sparteine. *Angew. Chem. Int. Ed.* **2018**, *57*, 223–226.

	Parameter	Value
1	Data File Name	/ Users/ jeffkerkovius/ Documents/ NMR Filess/ Other PPL NMR/ PHL1/ PHL1-149-C/ PROTON01.fid/ fid
2	Title	PROTON01
3	Origin	Varian
4	Instrument	inova
5	Solvent	cdcl3
6	Temperature	3.0
7	Pulse Sequence	s2pul
8	Experiment	1D
9	Number of Scans	8
10	Receiver Gain	60
11	Relaxation Delay	1.0000
12	Pulse Width	5.6500
13	Acquisition Time	3.0000
14	Acquisition Date	2021-11-10T16:49:44
15	Spectrometer Frequency	499.58
16	Spectral Width	8000.0
17	Lowest Frequency	-1018.6
18	Nucleus	1H
19	Acquired Size	24000
20	Spectral Size	65536

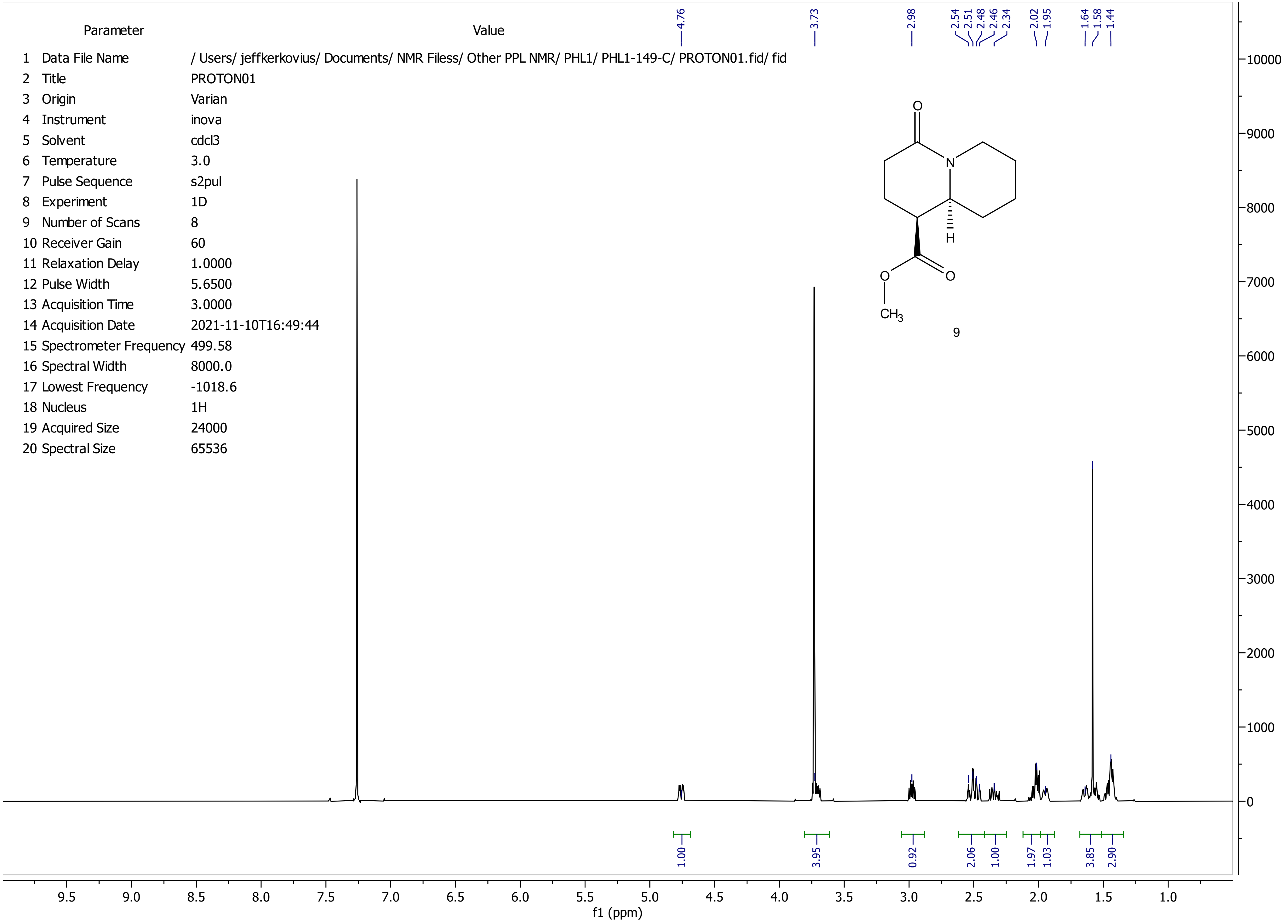

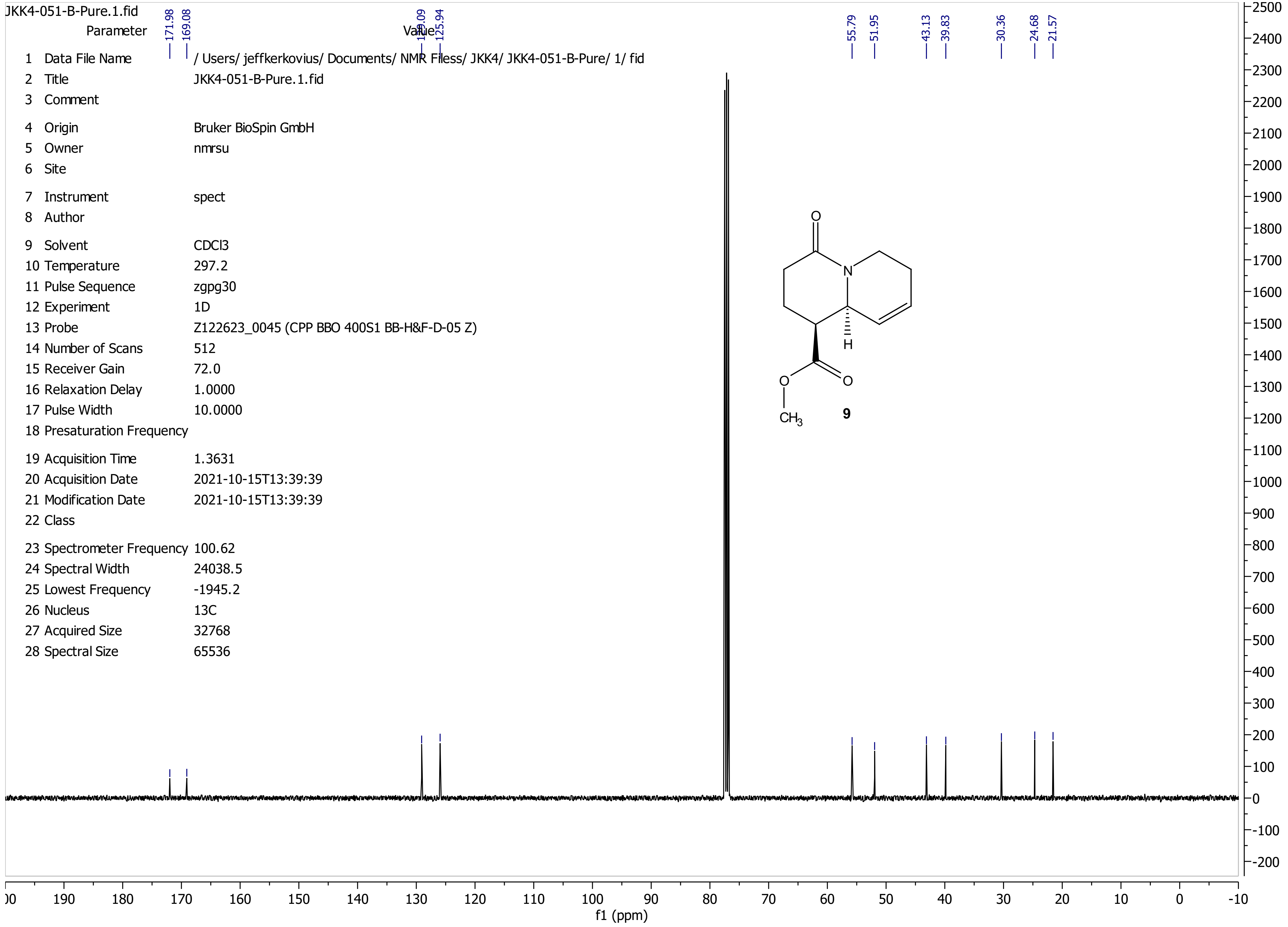

JKK4-051-B-Pure.1.fid

	Parameter	Value
1	Data File Name	/ Users/ jeffkerkovius/ Documents/ NMR Filess/ JKK4/ JKK4-051-B-Pure/ 1/ fid
2	Title	JKK4-051-B-Pure.1.fid
3	Comment	
4	Origin	Bruker BioSpin GmbH
5	Owner	nmrsu
6	Site	
7	Instrument	spect
8	Author	
9	Solvent	CDCl3
10	Temperature	297.2
11	Pulse Sequence	zgpg30
12	Experiment	1D
13	Probe	Z122623_0045 (CPP BBO 400S1 BB-H&F-D-05 Z)
14	Number of Scans	512
15	Receiver Gain	72.0
16	Relaxation Delay	1.0000
17	Pulse Width	10.0000
18	Presaturation Frequency	
19	Acquisition Time	1.3631
20	Acquisition Date	2021-10-15T13:39:39
21	Modification Date	2021-10-15T13:39:39
22	Class	
23	Spectrometer Frequency	100.62
24	Spectral Width	24038.5
25	Lowest Frequency	-1945.2
26	Nucleus	13C
27	Acquired Size	32768
28	Spectral Size	65536

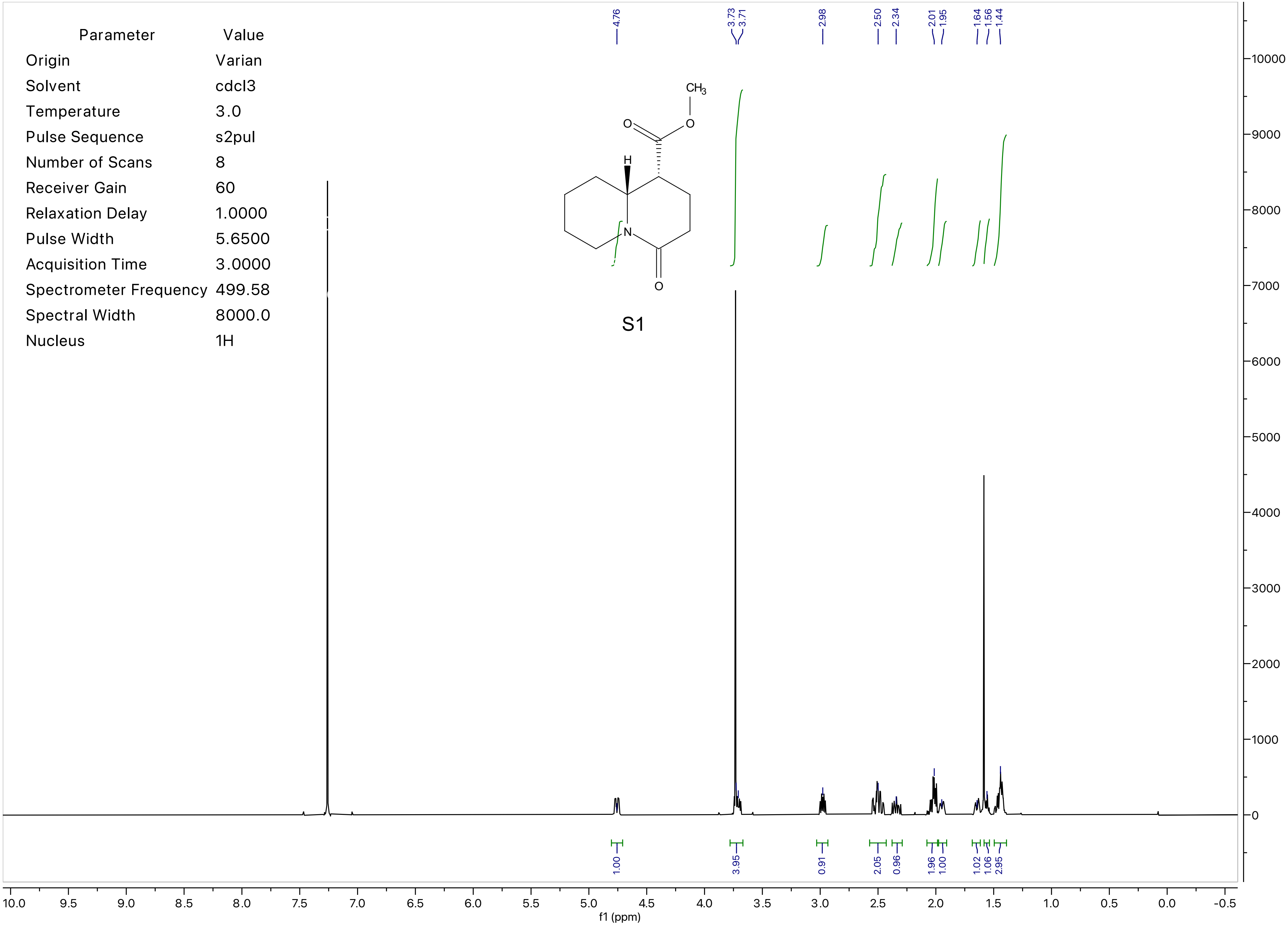

Parameter Value
Origin Varian
Solvent cdcl3
Temperature 3.0
Pulse Sequence s2pul
Number of Scans 8
Receiver Gain 60
Relaxation Delay 1.0000
Pulse Width 5.6500
Acquisition Time 3.0000
Spectrometer Frequency 499.58
Spectral Width 8000.0
Nucleus 1H
4.76
3.73
3.71
2.98
2.50
2.34
2.01
1.95
1.64
1.56
1.44
CH3
O
O
H
N
O
S1
1.00
3.95
0.91
2.05
0.96
1.96
1.00
1.02
1.06
2.95
f1 (ppm)

PHL1-149-X-Carbon.1.fid

	Parameter	Value
1	Data File Name	/ Users/ jeffkerkovius/ Documents/ NMR Filess/ Other PPL NMR/ PHL1/ PHL1-149-C-Carbon/ 1/ fid
2	Title	PHL1-149-X-Carbon.1.fid
3	Comment	
4	Origin	Bruker BioSpin GmbH
5	Owner	nmrsu
6	Site	
7	Instrument	spect
8	Author	
9	Solvent	CDCl3
10	Temperature	297.1
11	Pulse Sequence	zgpg30
12	Experiment	1D
13	Probe	Z122623_0045 (CPP BBO 400S1 BB-H&F-D-05 Z)
14	Number of Scans	512
15	Receiver Gain	72.0
16	Relaxation Delay	1.0000
17	Pulse Width	10.0000
18	Presaturation Frequency	
19	Acquisition Time	1.3631
20	Acquisition Date	2021-11-11T17:08:08
21	Modification Date	2021-11-11T17:08:08
22	Class	
23	Spectrometer Frequency	100.62
24	Spectral Width	24038.5
25	Lowest Frequency	-1945.0
26	Nucleus	13C
27	Acquired Size	32768
28	Spectral Size	65536

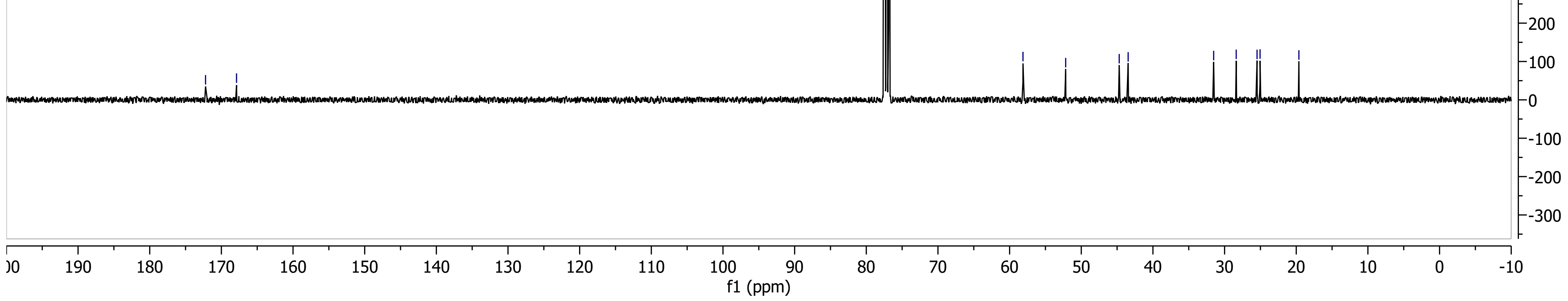

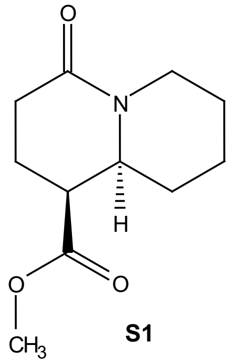

S1

PHL2-069-C-PROTON_01
PHL2-069-C

	Parameter	Value
1	Data File Name	Y:/ pllam/ vnmrsys/ data/ PHL2-069-C_20230221_01/ PHL2-069-C-PROTON_01.fid/ fid
2	Title	PHL2-069-C-PROTON_01
3	Comment	PHL2-069-C
4	Origin	Varian
5	Owner	
6	Site	
7	Instrument	inova
8	Author	
9	Solvent	cdcl3
10	Temperature	25.0
11	Pulse Sequence	s2pul
12	Experiment	1D
13	Probe	penta
14	Number of Scans	8
15	Receiver Gain	28
16	Relaxation Delay	1.0000
17	Pulse Width	2.6500
18	Presaturation Frequency	
19	Acquisition Time	1.7046
20	Acquisition Date	2023-02-21T15:52:20
21	Modification Date	2023-02-21T15:52:51
22	Class	
23	Spectrometer Frequency	599.56
24	Spectral Width	9611.9
25	Lowest Frequency	-1197.9
26	Nucleus	1H
27	Acquired Size	16384
28	Spectral Size	65536
29	Digital Resolution	0.15

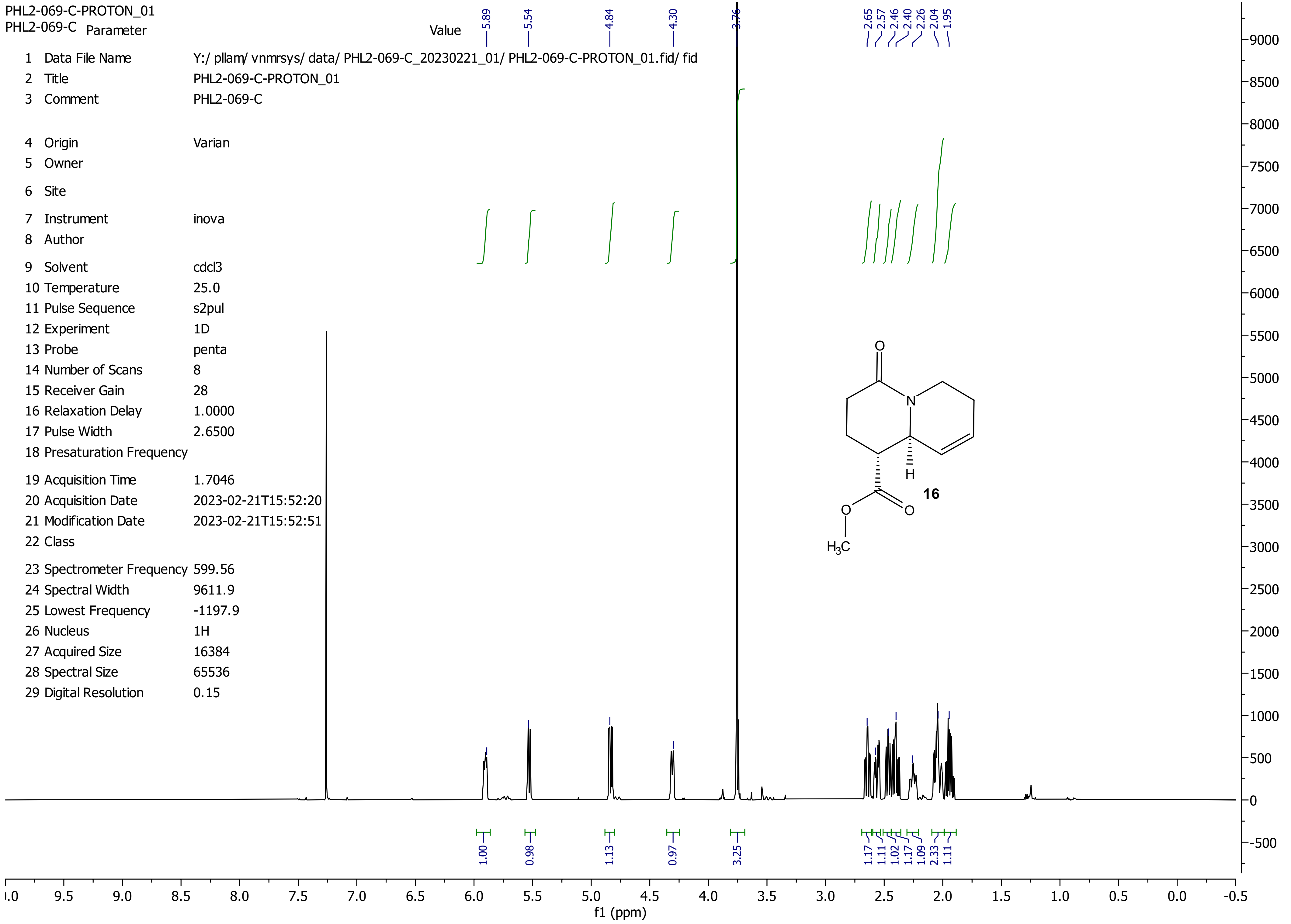

16

PHL2-069-C-Carbon.3.fid

	Parameter	Value
1	Data File Name	X:/ pllam/ nmr/ PHL2-069-C-Carbon/ 3/ fid
2	Title	PHL2-069-C-Carbon.3.fid
3	Comment	
4	Origin	Bruker BioSpin GmbH
5	Owner	nmrsu
6	Site	
7	Instrument	spect
8	Author	
9	Solvent	CDCl3
10	Temperature	297.1
11	Pulse Sequence	zgpg30
12	Experiment	1D
13	Probe	Z122623_0045 (CPP BBO 400S1 BB-H&F-D-05 Z)
14	Number of Scans	512
15	Receiver Gain	72.0
16	Relaxation Delay	1.0000
17	Pulse Width	10.0000
18	Presaturation Frequency	
19	Acquisition Time	1.3631
20	Acquisition Date	2023-02-21T16:47:05
21	Modification Date	2023-02-21T16:47:05
22	Class	
23	Spectrometer Frequency	100.62
24	Spectral Width	24038.5
25	Lowest Frequency	-1914.0
26	Nucleus	13C
27	Acquired Size	32768
28	Spectral Size	65536
29	Digital Resolution	0.37

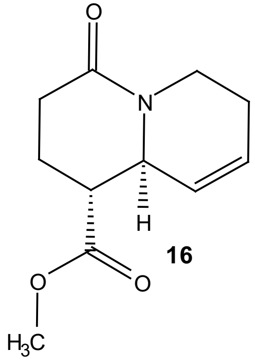

16

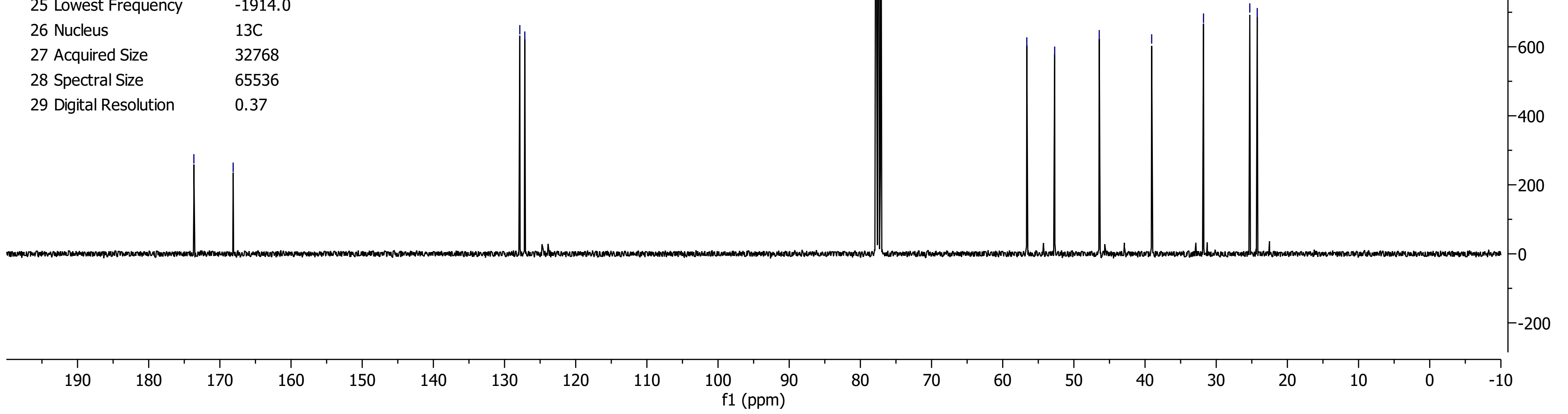

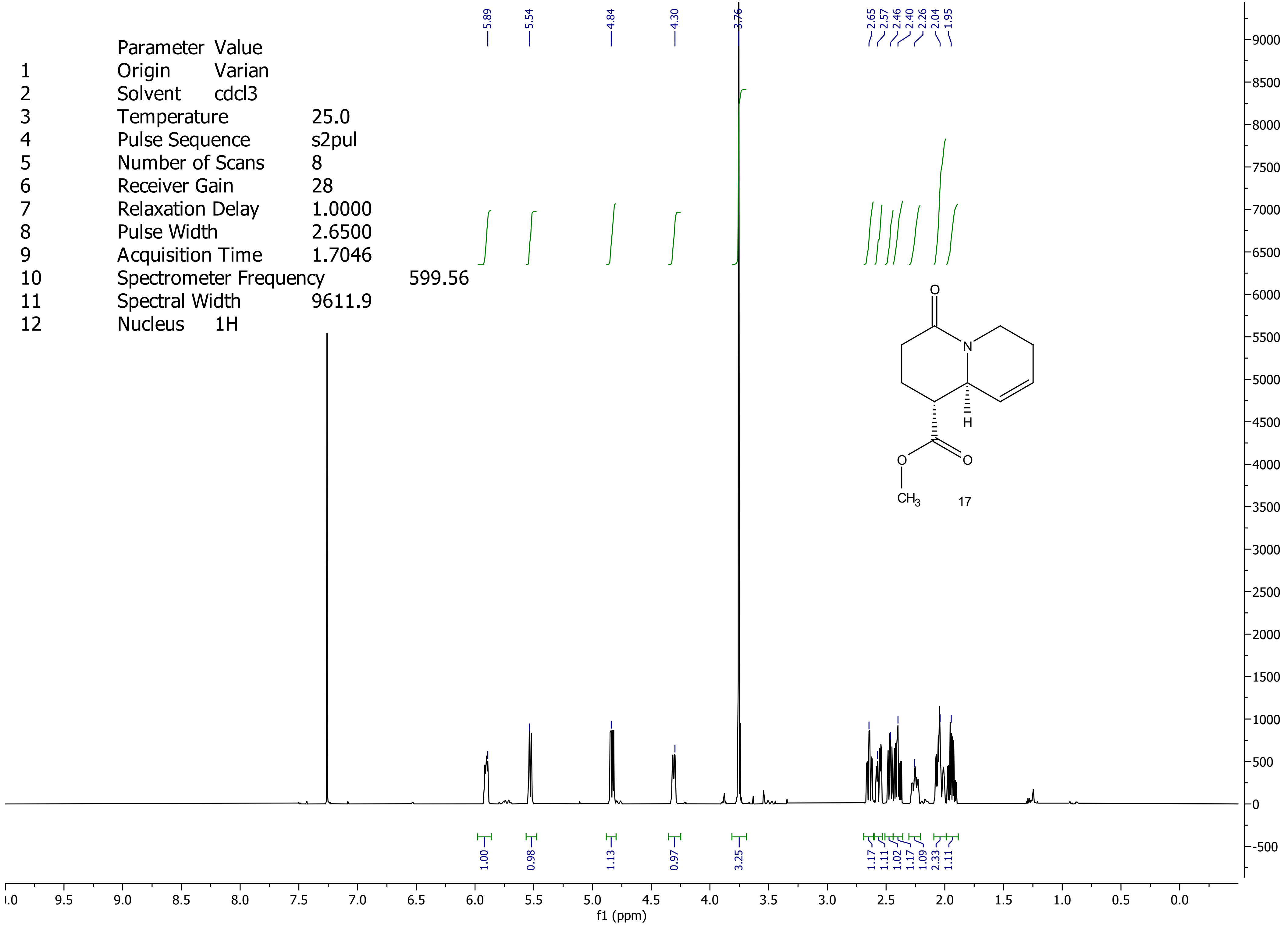

Parameter Value
1 Origin Varian
2 Solvent cdcl3
3 Temperature 25.0
4 Pulse Sequence s2pul
5 Number of Scans 8
6 Receiver Gain 28
7 Relaxation Delay 1.0000
8 Pulse Width 2.6500
9 Acquisition Time 1.7046
10 Spectrometer Frequency 599.56
11 Spectral Width 9611.9
12 Nucleus 1H
5.89
5.54
4.84
4.30
3.76
2.65
2.57
2.46
2.40
2.26
2.04
1.95
1.00
0.98
1.13
0.97
3.25
1.17
1.11
1.02
1.17
1.09
2.33
1.11
O
N
H
O
O
CH3
17
f1 (ppm)

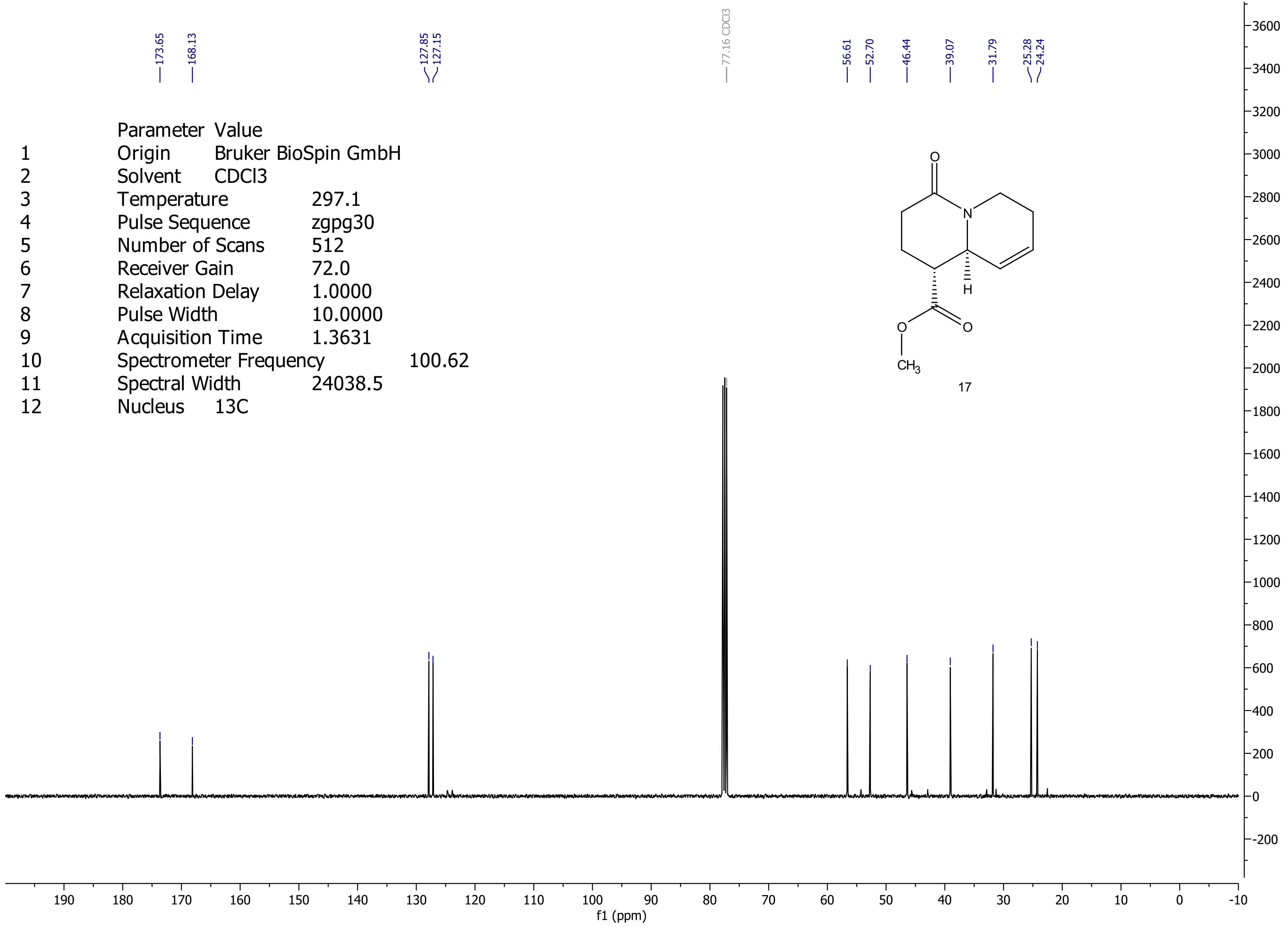

	Parameter	Value
1	Origin	Bruker BioSpin GmbH
2	Solvent	CDCl3
3	Temperature	297.1
4	Pulse Sequence	zgpg30
5	Number of Scans	512
6	Receiver Gain	72.0
7	Relaxation Delay	1.0000
8	Pulse Width	10.0000
9	Acquisition Time	1.3631
10	Spectrometer Frequency	100.62
11	Spectral Width	24038.5
12	Nucleus	13C

	Parameter	Value
1	Origin	Varian
2	Solvent	cdcl3
3	Temperature	25.0
4	Pulse Sequence	s2pul
5	Number of Scans	8
6	Receiver Gain	26
7	Relaxation Delay	1.0000
8	Pulse Width	2.6500
9	Acquisition Time	1.7046
10	Spectrometer Frequency	599.56
11	Spectral Width	9611.9
12	Nucleus	1H

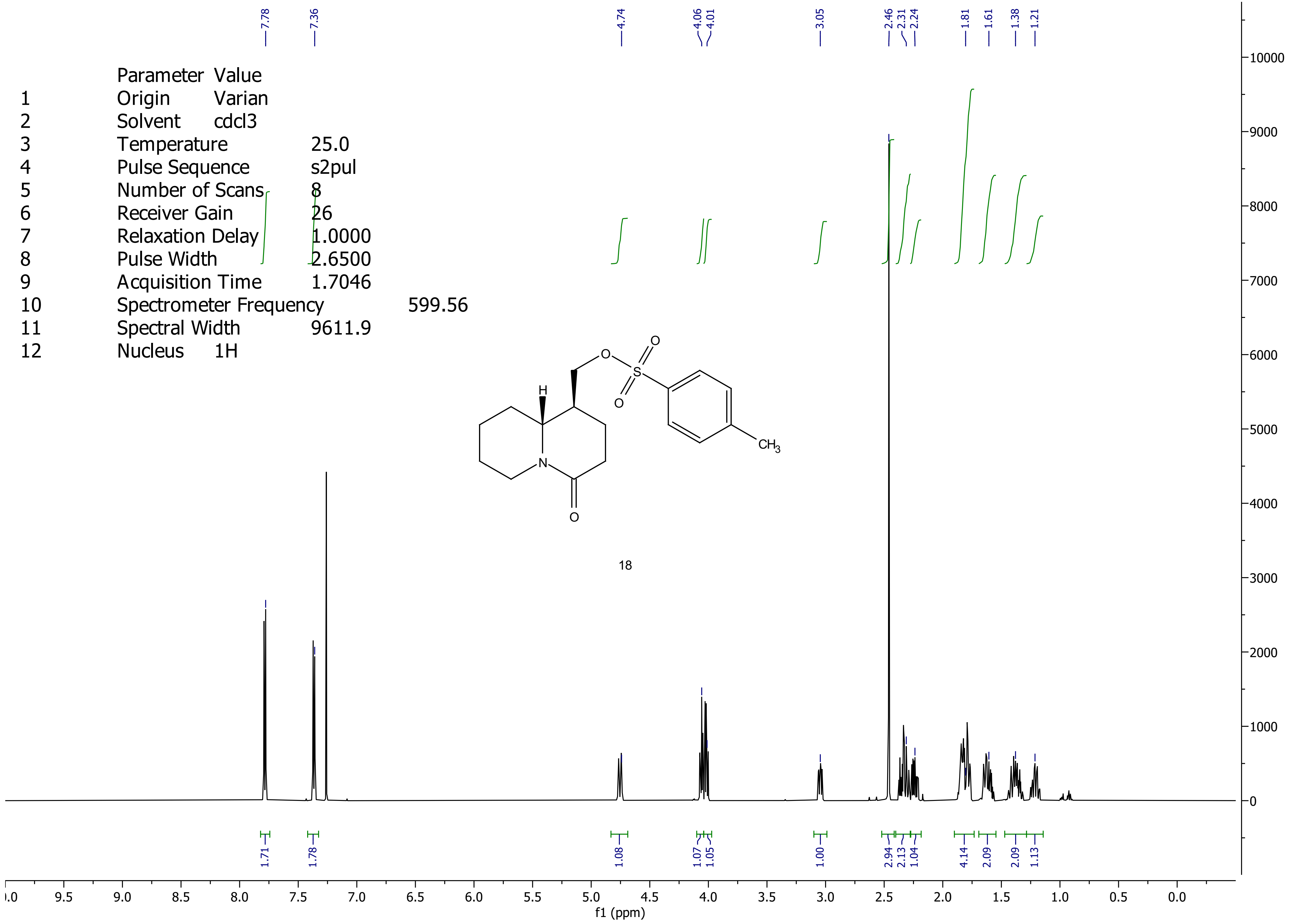

18

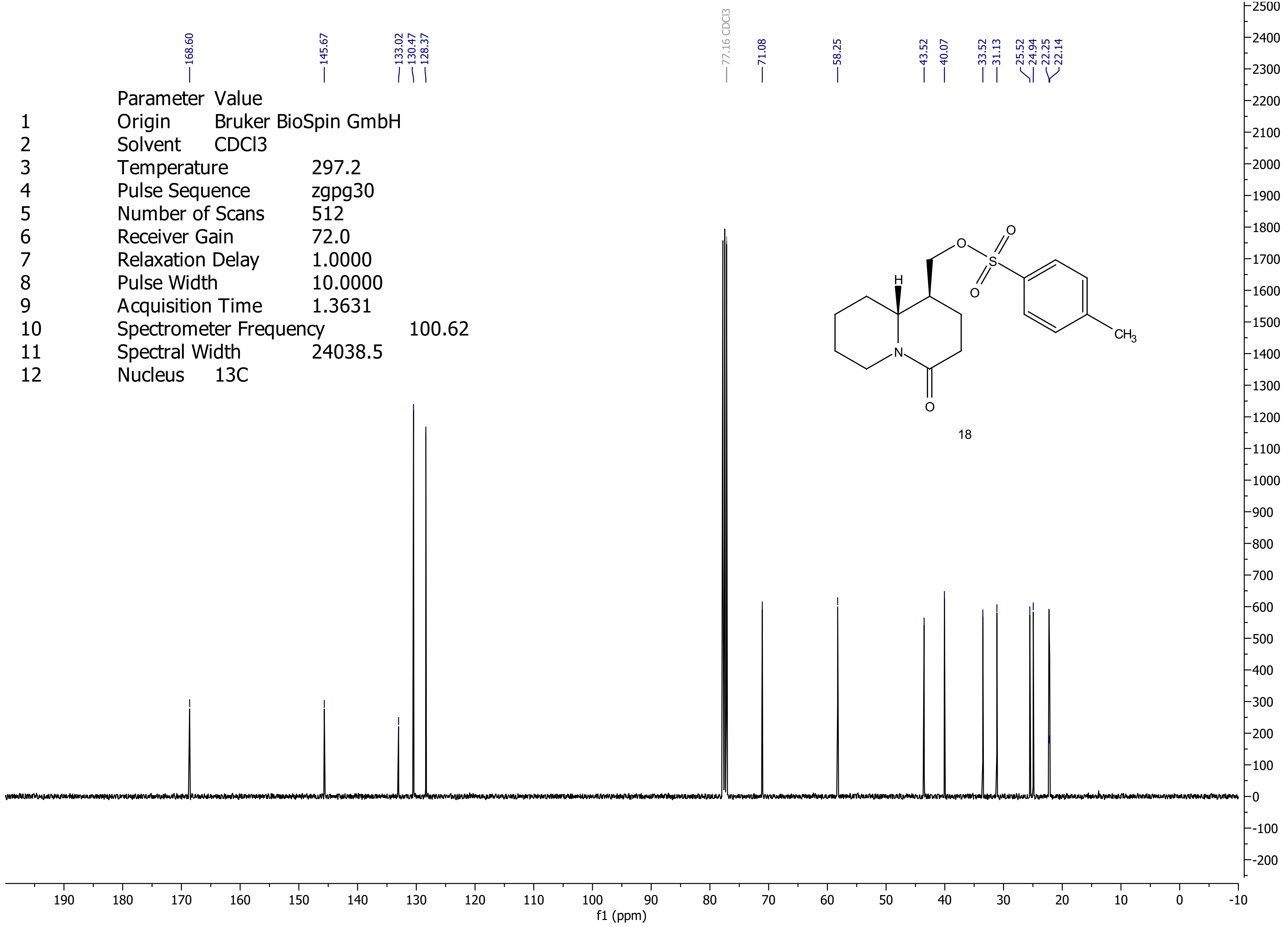

168.60
145.67
133.02
130.47
128.37
77.16 CDCl3
71.08
58.25
43.52
40.07
33.52
31.13
25.52
24.94
22.25
22.14
Parameter Value
1 Origin Bruker BioSpin GmbH
2 Solvent CDCl3
3 Temperature 297.2
4 Pulse Sequence zgpg30
5 Number of Scans 512
6 Receiver Gain 72.0
7 Relaxation Delay 1.0000
8 Pulse Width 10.0000
9 Acquisition Time 1.3631
10 Spectrometer Frequency 100.62
11 Spectral Width 24038.5
12 Nucleus 13C
H
N
O
S
CH3
18
f1 (ppm)

PHL2-074-D-Attempt2-PROTON_01
PHL2-074-D-Attempt2

	Parameter	Value
1	Data File Name	Y:/ pllam/ vnmrsys/ data/ PHL2-074-D-Attempt2_20230227_01/ PHL2-074-D-Attempt2-PROTON_01.fid/ fid
2	Title	PHL2-074-D-Attempt2-PROTON_01
3	Comment	PHL2-074-D-Attempt2
4	Origin	Varian
5	Owner	
6	Site	
7	Instrument	inova
8	Author	
9	Solvent	cdcl3
10	Temperature	25.0
11	Pulse Sequence	s2pul
12	Experiment	1D
13	Probe	penta
14	Number of Scans	8
15	Receiver Gain	40
16	Relaxation Delay	1.0000
17	Pulse Width	2.6500
18	Presaturation Frequency	
19	Acquisition Time	1.7046
20	Acquisition Date	2023-02-27T15:19:19
21	Modification Date	2023-02-27T15:19:50
22	Class	
23	Spectrometer Frequency	599.56
24	Spectral Width	9611.9
25	Lowest Frequency	-1198.1
26	Nucleus	1H
27	Acquired Size	16384
28	Spectral Size	65536
29	Digital Resolution	0.15

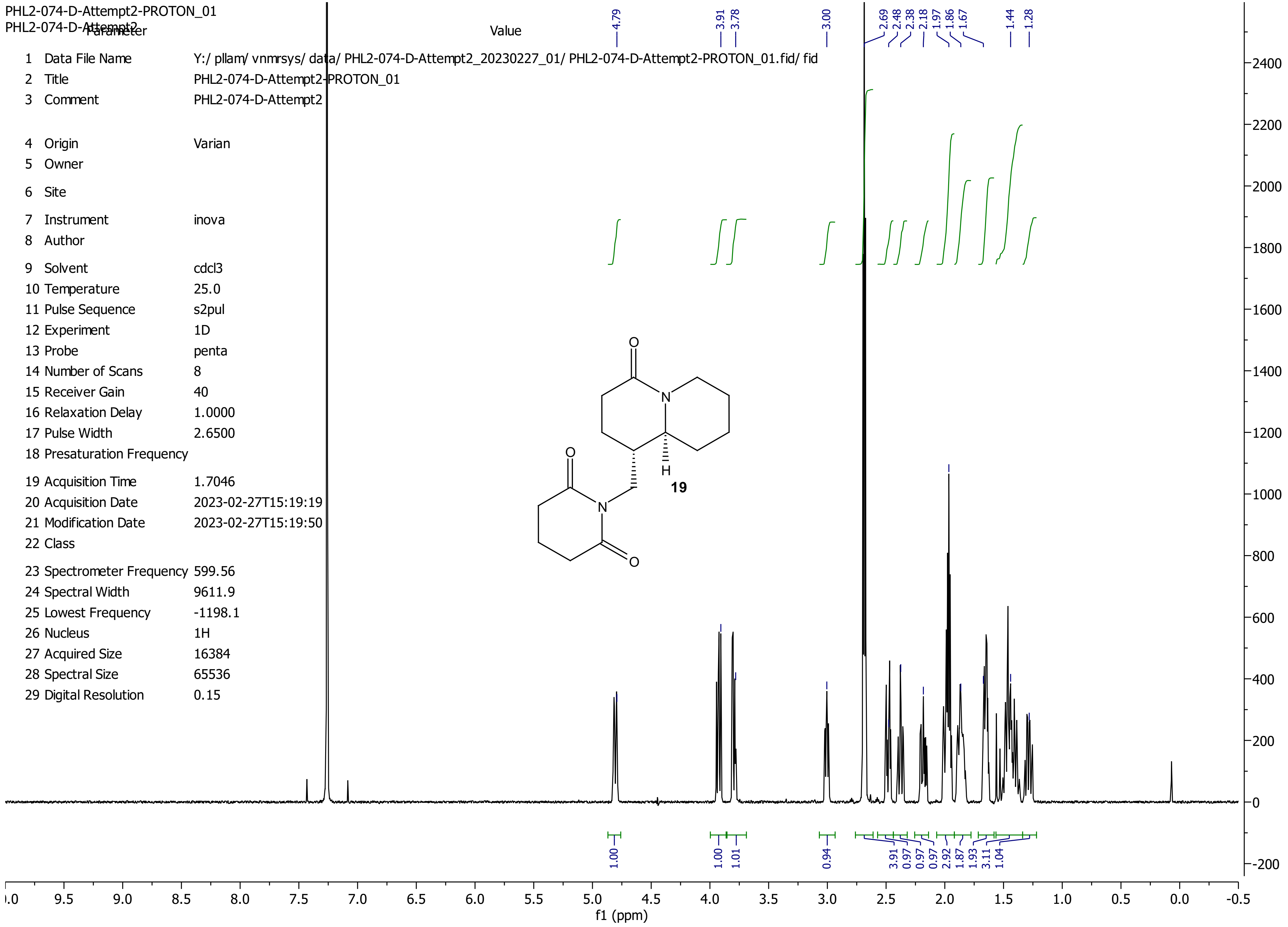

PHL2-074-D-Carbon-Attempt2.3.fid

	Parameter	Value
1	Data File Name	X:/ pllam/ nmr/ PHL2-074-D-Carbon-Attempt2/ 3/ fid
2	Title	PHL2-074-D-Carbon-Attempt2.3.fid
3	Comment	
4	Origin	Bruker BioSpin GmbH
5	Owner	nmrsu
6	Site	
7	Instrument	spect
8	Author	
9	Solvent	CDCl3
10	Temperature	297.2
11	Pulse Sequence	zgpg30
12	Experiment	1D
13	Probe	Z122623_0045 (CPP BBO 400S1 BB-H&F-D-05 Z)
14	Number of Scans	512
15	Receiver Gain	64.2
16	Relaxation Delay	1.0000
17	Pulse Width	10.0000
18	Presaturation Frequency	
19	Acquisition Time	1.3631
20	Acquisition Date	2023-02-27T16:22:04
21	Modification Date	2023-02-27T16:22:04
22	Class	
23	Spectrometer Frequency	100.62
24	Spectral Width	24038.5
25	Lowest Frequency	-1914.0
26	Nucleus	13C
27	Acquired Size	32768
28	Spectral Size	65536
29	Digital Resolution	0.37

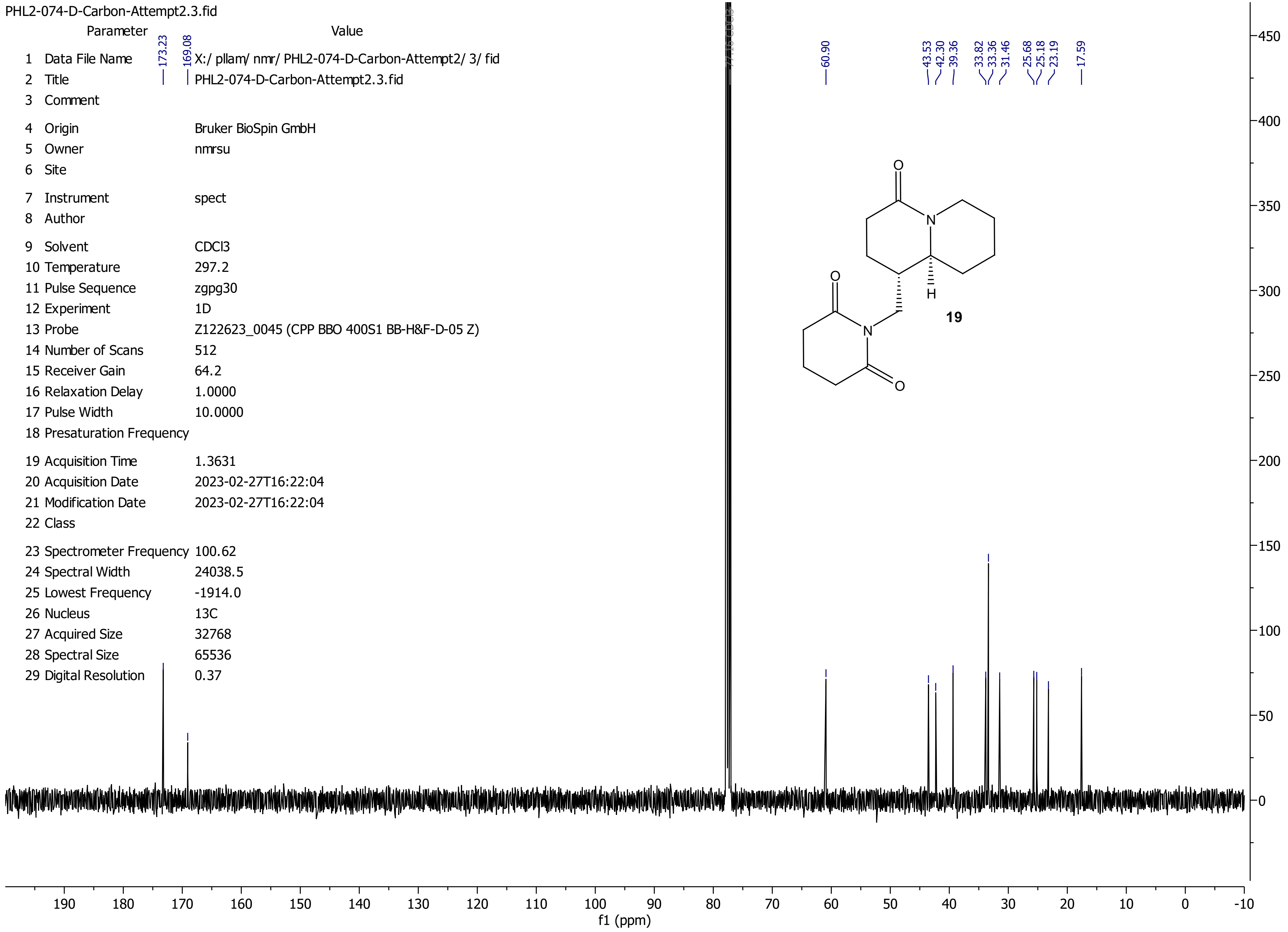

PHL2-075-B-PROTON_01
PHL2-075-B

	Parameter	Value
1	Data File Name	Y:/ pllam/ vnmrsys/ data/ PHL2-075-B_20230227_01/ PHL2-075-B-PROTON_01.fid/ fid
2	Title	PHL2-075-B-PROTON_01
3	Comment	PHL2-075-B
4	Origin	Varian
5	Owner	
6	Site	
7	Instrument	inova
8	Author	
9	Solvent	cdcl3
10	Temperature	25.0
11	Pulse Sequence	s2pul
12	Experiment	1D
13	Probe	penta
14	Number of Scans	8
15	Receiver Gain	26
16	Relaxation Delay	1.0000
17	Pulse Width	2.6500
18	Presaturation Frequency	
19	Acquisition Time	1.7046
20	Acquisition Date	2023-02-27T16:52:07
21	Modification Date	2023-02-27T16:52:38
22	Class	
23	Spectrometer Frequency	599.56
24	Spectral Width	9611.9
25	Lowest Frequency	-1198.0
26	Nucleus	1H
27	Acquired Size	16384
28	Spectral Size	65536
29	Digital Resolution	0.15

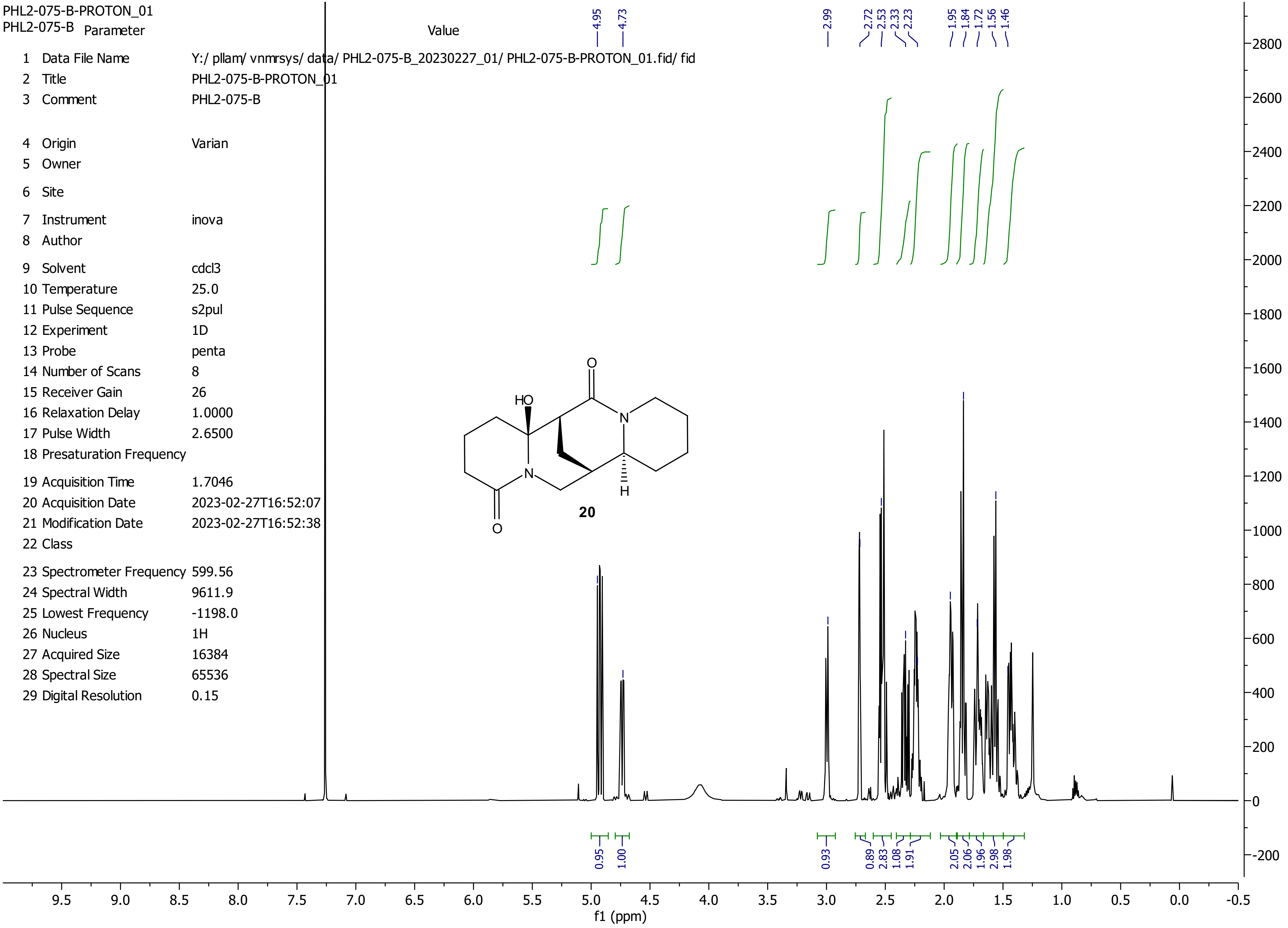

PHL2-075-B-Carbon.3.fid

	Parameter	Value
1	Data File Name	X:/ pllam/ nmr/ PHL2-075-B-Carbon/ 3/ fid
2	Title	PHL2-075-B-Carbon.3.fid
3	Comment	
4	Origin	Bruker BioSpin GmbH
5	Owner	nmrsu
6	Site	
7	Instrument	spect
8	Author	
9	Solvent	CDCl3
10	Temperature	297.2
11	Pulse Sequence	zgpg30
12	Experiment	1D
13	Probe	Z122623_0045 (CPP BBO 400S1 BB-H&F-D-05 Z)
14	Number of Scans	512
15	Receiver Gain	64.2
16	Relaxation Delay	1.0000
17	Pulse Width	10.0000
18	Presaturation Frequency	
19	Acquisition Time	1.3631
20	Acquisition Date	2023-02-27T17:26:48
21	Modification Date	2023-02-27T17:26:48
22	Class	
23	Spectrometer Frequency	100.62
24	Spectral Width	24038.5
25	Lowest Frequency	-1914.0
26	Nucleus	13C
27	Acquired Size	32768
28	Spectral Size	65536
29	Digital Resolution	0.37

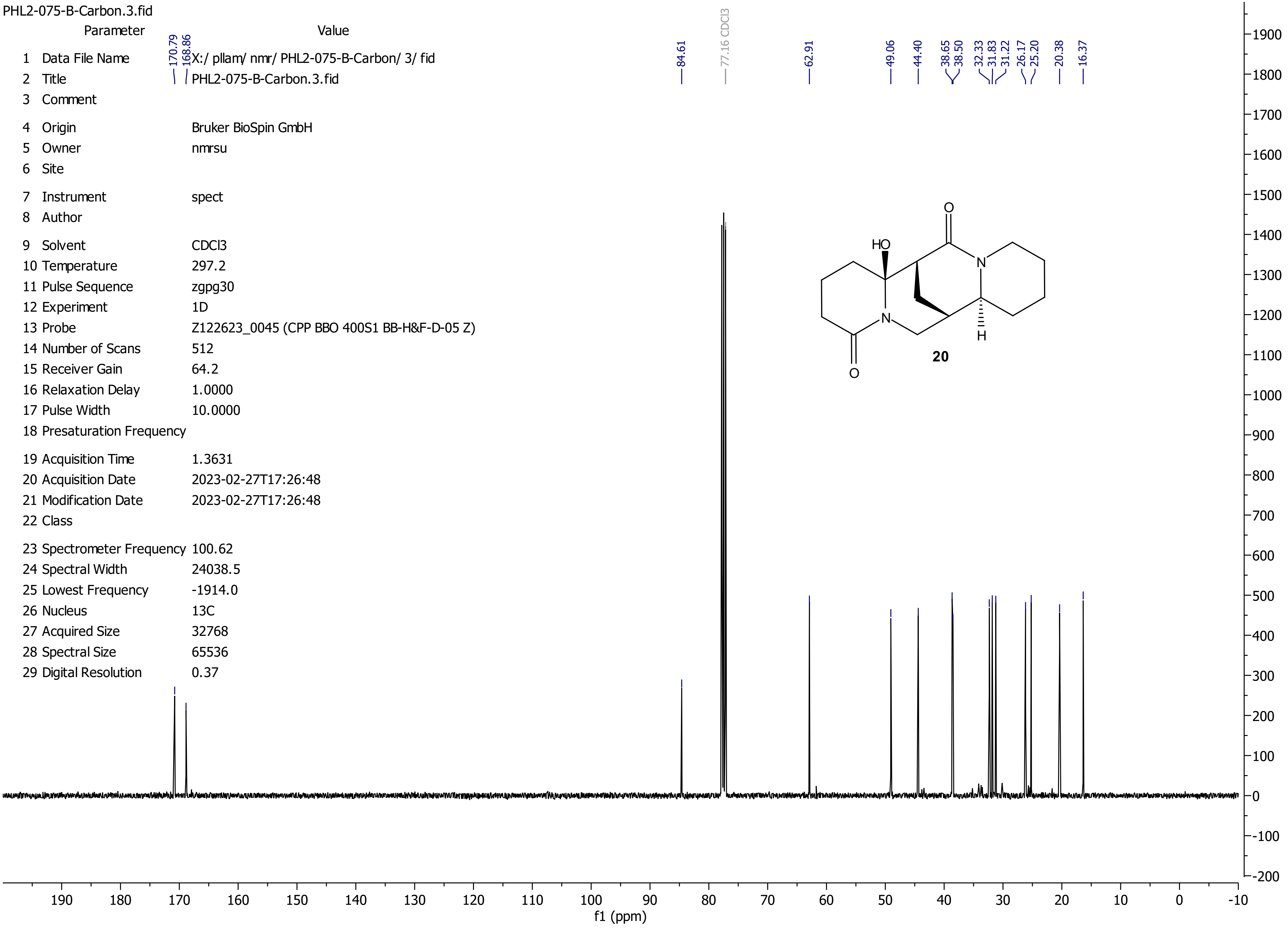

20

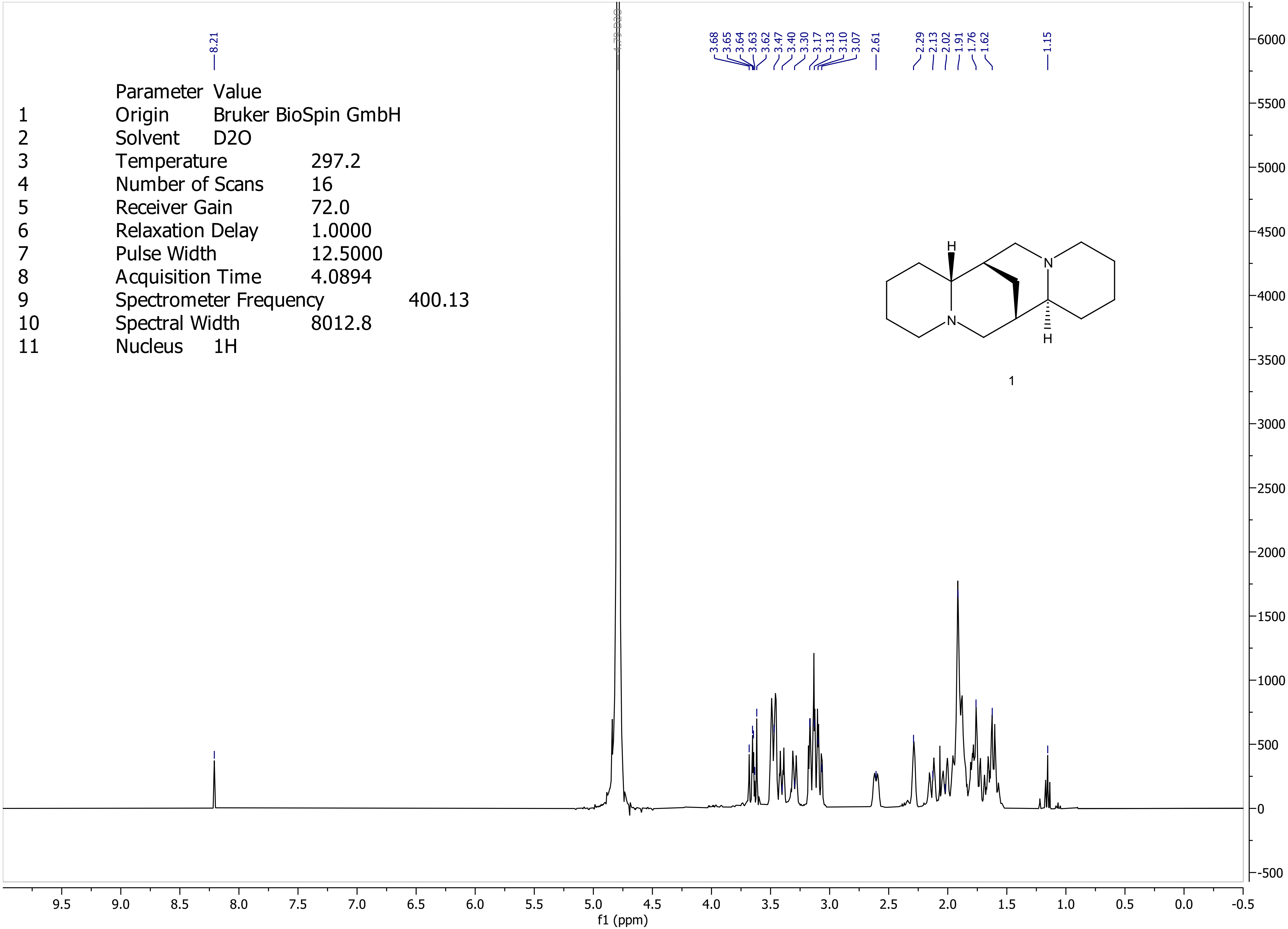

8.21
3.68
3.65
3.64
3.63
3.62
3.47
3.40
3.30
3.17
3.13
3.10
3.07
2.61
2.29
2.13
2.02
1.91
1.76
1.62
1.15
Parameter Value
1 Origin Bruker BioSpin GmbH
2 Solvent D2O
3 Temperature 297.2
4 Number of Scans 16
5 Receiver Gain 72.0
6 Relaxation Delay 1.0000
7 Pulse Width 12.5000
8 Acquisition Time 4.0894
9 Spectrometer Frequency 400.13
10 Spectral Width 8012.8
11 Nucleus 1H
H
N
N
H
1
f1 (ppm)

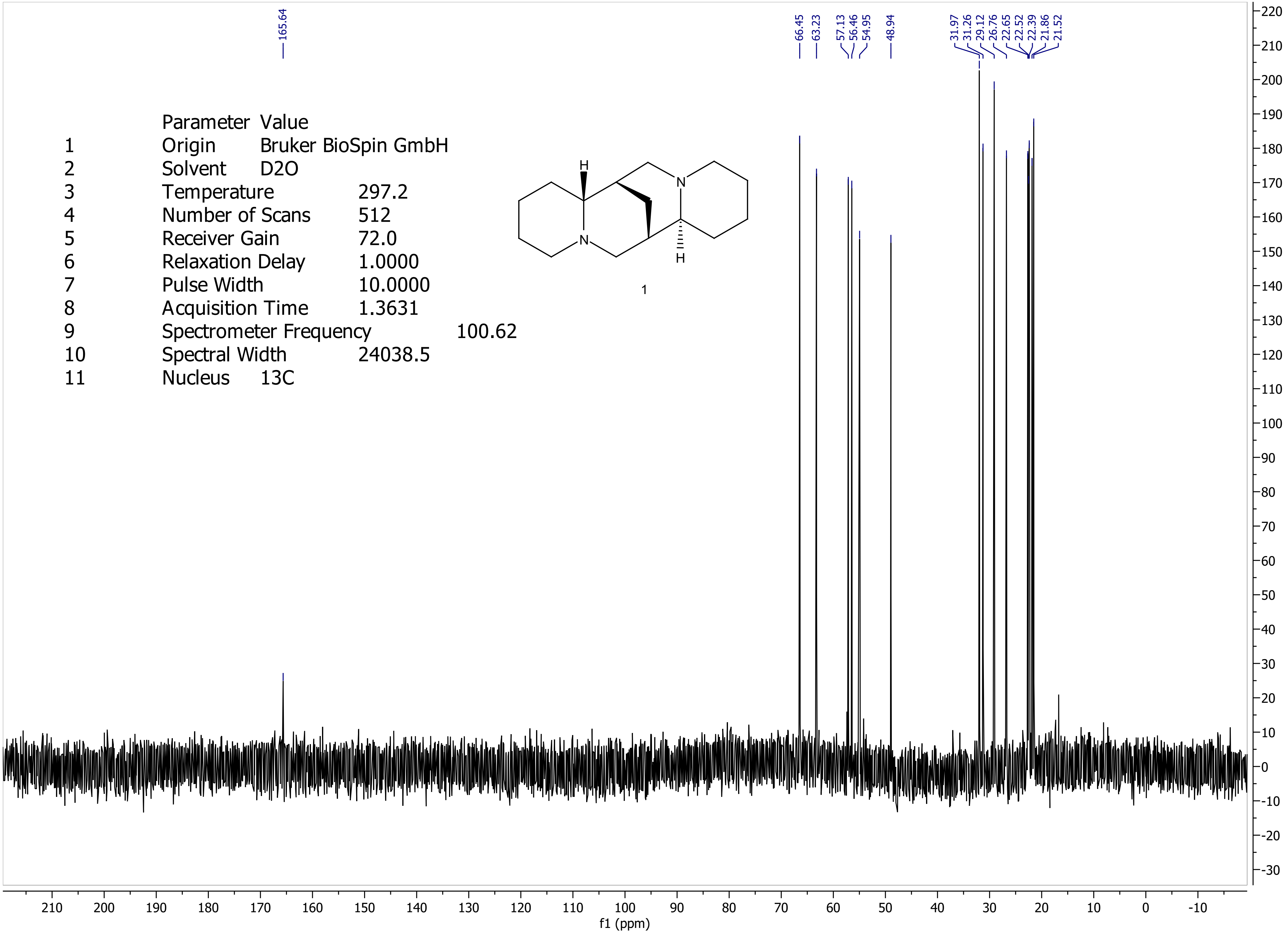
165.64
66.45
63.23
57.13
56.46
54.95
48.94
31.97
31.26
29.12
26.76
22.65
22.52
22.39
21.86
21.52
Parameter Value
1 Origin Bruker BioSpin GmbH
2 Solvent D2O
3 Temperature 297.2
4 Number of Scans 512
5 Receiver Gain 72.0
6 Relaxation Delay 1.0000
7 Pulse Width 10.0000
8 Acquisition Time 1.3631
9 Spectrometer Frequency 100.62
10 Spectral Width 24038.5
11 Nucleus 13C
H
N
N
H
1
f1 (ppm)

PHL2-086-Xs-PROTON_01
PHL2-086-Xs

Parameter	Value
1 Solvent	d2o
2 Temperature	25.0
3 Pulse Sequence	s2pul
4 Experiment	1D
5 Probe	penta
6 Number of Scans	8
7 Spectrometer Frequency	599.56
8 Nucleus	1H

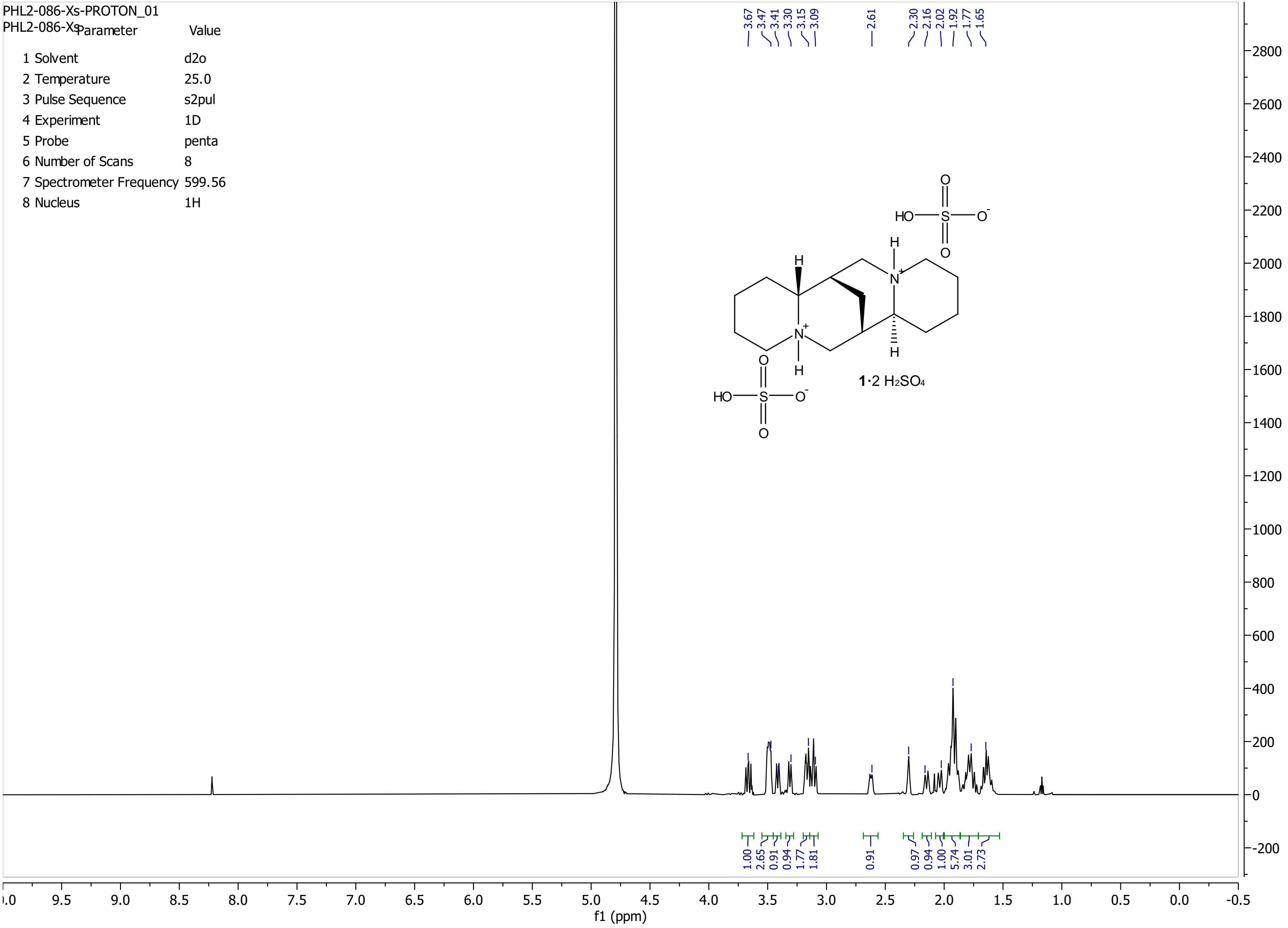

PHL2-086-X-Carbon.1.fid

	Parameter	Value
1	Solvent	D2O
2	Temperature	297.2
3	Pulse Sequence	zgpg30
4	Experiment	1D
5	Probe	Z122623_0045 (CPP BBO 400S1 BB-H&F-D-05 Z)
6	Number of Scans	512
7	Spectrometer Frequency	100.62
8	Nucleus	13C

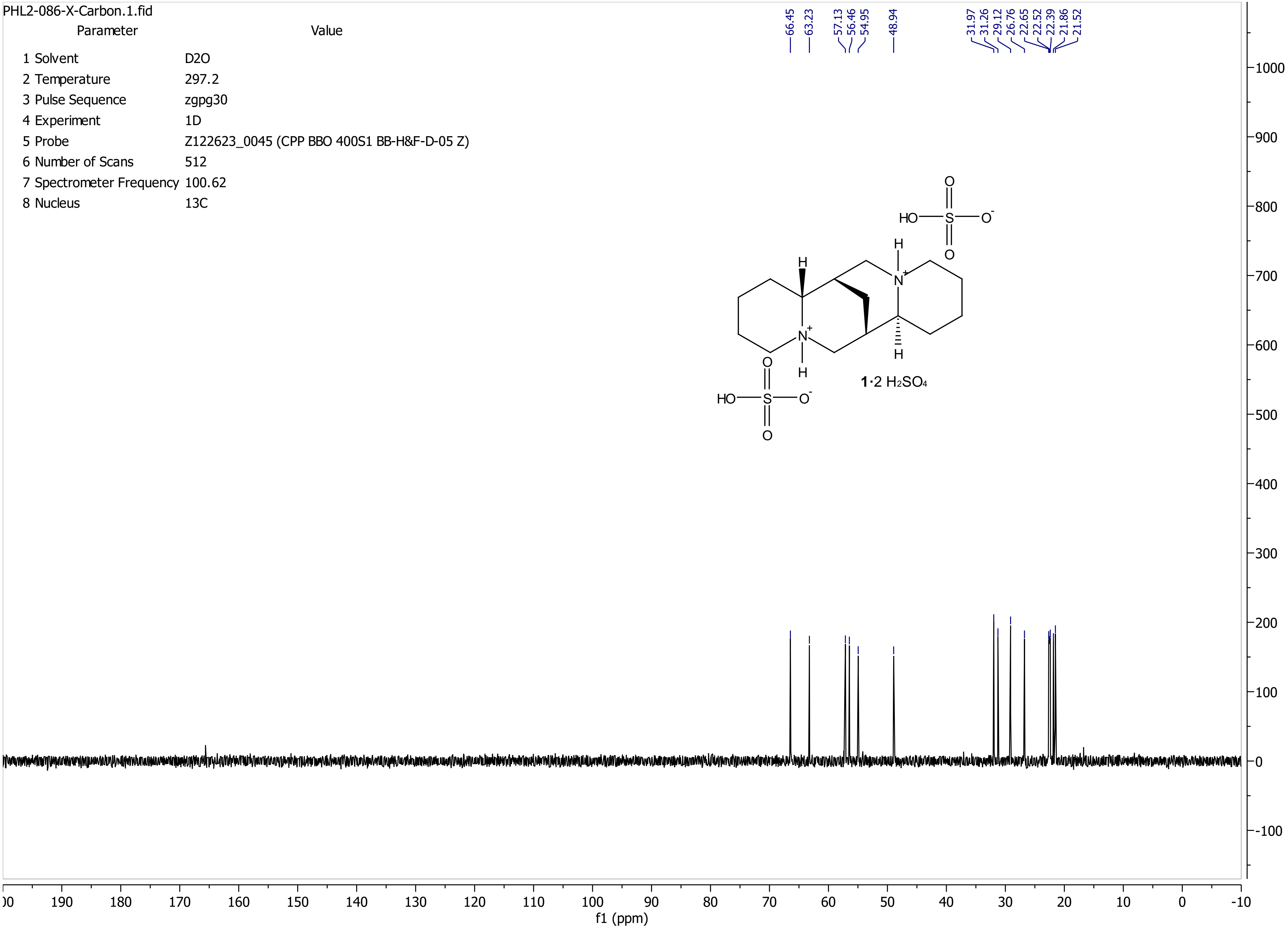

Parameter	Value
1 Data File Name	/ Users/ jeffkerkovius/ Documents/ NMR Filess/ Other PPL NMR/ PHL1/ PHL1-151-B-Purer/ PROTON01.fid/ fid
2 Title	PROTON01
3 Origin	Varian
4 Instrument	inova
5 Solvent	cdcl3
6 Temperature	3.0
7 Pulse Sequence	s2pul
8 Experiment	1D
9 Number of Scans	8
10 Receiver Gain	44
11 Relaxation Delay	1.0000
12 Pulse Width	5.6500
13 Acquisition Time	3.0000
14 Acquisition Date	2021-11-15T14:21:11
15 Spectrometer Frequency	499.58
16 Spectral Width	8000.0
17 Lowest Frequency	-1018.6
18 Nucleus	1H
19 Acquired Size	24000
20 Spectral Size	65536

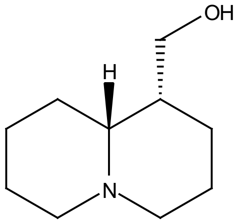

21

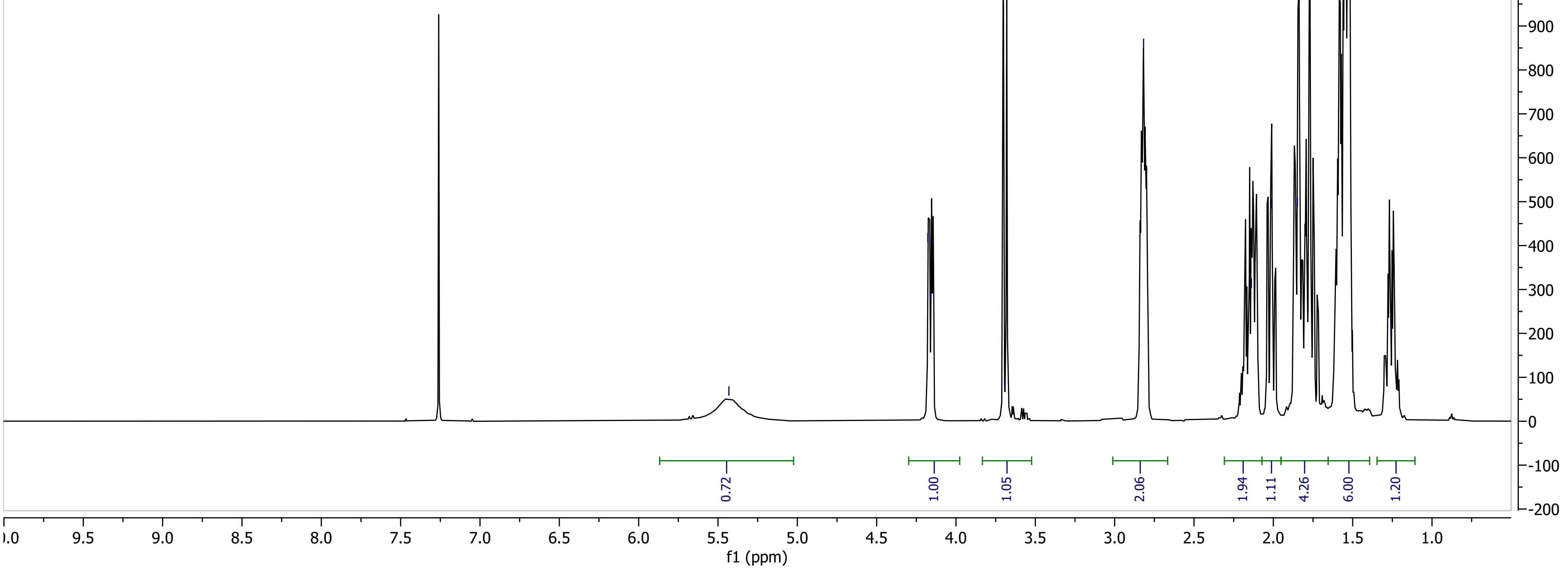

PHL1-151-B-Carbon.1.fid

	Parameter	Value
1	Data File Name	/ Users/ jeffkerkovius/ Documents/ NMR Filess/ Other PPL NMR/ PHL1/ PHL1-151-B-Carbon/ 1/ fid
2	Title	PHL1-151-B-Carbon.1.fid
3	Comment	
4	Origin	Bruker BioSpin GmbH
5	Owner	nmrsu
6	Site	
7	Instrument	spect
8	Author	
9	Solvent	CDCl3
10	Temperature	297.2
11	Pulse Sequence	zgpg30
12	Experiment	1D
13	Probe	Z122623_0045 (CPP BBO 400S1 BB-H&F-D-05 Z)
14	Number of Scans	512
15	Receiver Gain	50.3
16	Relaxation Delay	1.0000
17	Pulse Width	10.0000
18	Presaturation Frequency	
19	Acquisition Time	1.3631
20	Acquisition Date	2021-11-15T17:15:34
21	Modification Date	2021-11-15T17:15:34
22	Class	
23	Spectrometer Frequency	100.62
24	Spectral Width	24038.5
25	Lowest Frequency	-1945.9
26	Nucleus	13C
27	Acquired Size	32768
28	Spectral Size	65536

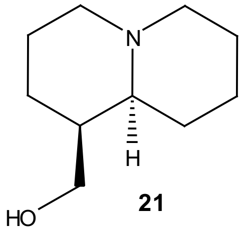

21

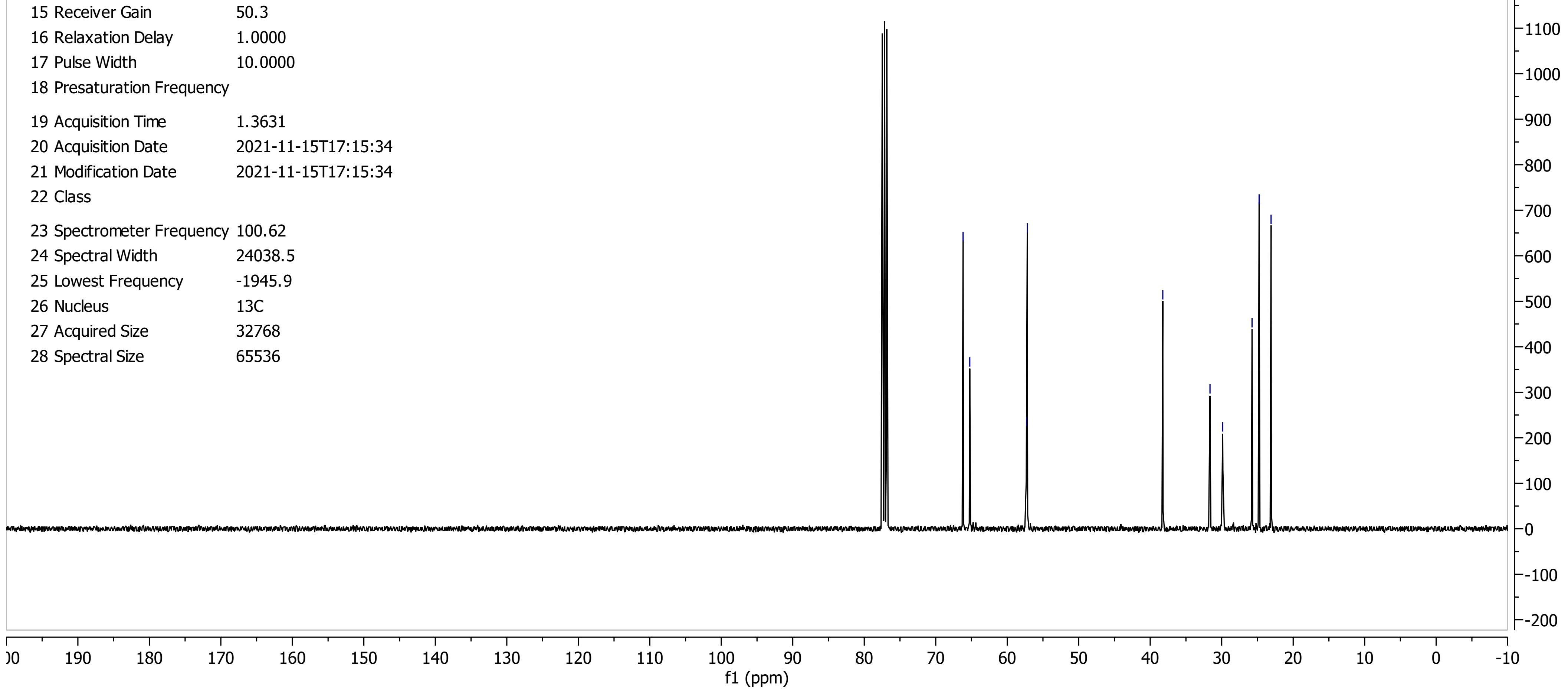

Supplement: Supplementary file 1 — ol3c03242_si_001.pdf [file ol3c03242_si_001.pdf]
